# Specific cell subclusters of dental pulp stem cells respond to distinct pathogens through the ROS pathway

**DOI:** 10.3389/fcimb.2024.1452124

**Published:** 2024-09-12

**Authors:** Tiansong Xu, Yangjia Liu, Wen Zhang, Murong Li, Liqi Zhang, Xueying Li, Yifei Zhang, Lin Yue, Sha Li, Ye Lin, Xiaoying Zou, Feng Chen

**Affiliations:** ^1^ Central Laboratory, Peking University School and Hospital of Stomatology & National Center for Stomatology & National Clinical Research Center for Oral Diseases & National Engineering Research Center of Oral Biomaterials and Digital Medical Devices, Beijing, China; ^2^ Fifth Clinical Division, Peking University School and Hospital of Stomatology & National Center for Stomatology & National Clinical Research Center for Oral Diseases & National Engineering Research Center of Oral Biomaterials and Digital Medical Devices, Beijing, China; ^3^ Department of Cariology and Endodontology, Peking University School and Hospital of Stomatology & National Center for Stomatology & National Clinical Research Center for Oral Diseases & National Engineering Research Center of Oral Biomaterials and Digital Medical Devices, Beijing, China; ^4^ Department of Stomatology, Peking University International Hospital, Beijing, China; ^5^ Department of Implantology, Peking University School and Hospital of Stomatology & National Center for Stomatology & National Clinical Research Center for Oral Diseases & National Engineering Research Center of Oral Biomaterials and Digital Medical Devices, Beijing, China; ^6^ Center of Stomatology, Peking University Hospital, Beijing, China

**Keywords:** dental pulp stem cells, *Candida albicans*, MAPK/ERK1/2, FOS, immune response

## Abstract

**Introduction:**

Microbial pathogens invade various human organs, including the oral cavity. *Candida albicans* (C.a) and *Streptococcus mutans* (S.m) served respectively as representative oral pathogenic fungi and bacteria to stimulate dental pulp stem cells (DPSCs) and to screen the DPSC subcluster that specifically responded to fungal infection.

**Methods:**

DPSCs were obtained from the impacted third molars of six healthy subjects. Then, cells were mixed and divided into three samples, two of which were stimulated with C.a and S.m, respectively; the third sample was exposed to cell medium only (Ctrl). Single-cell mRNA sequencing analysis of treated DPSCs was performed.

**Results:**

DPSCs were composed of four major clusters of which one, DPSC.7, exhibited unique changes compared to those of other subclusters. The DPSC.7 cell percentage of the C.a sample was twice those of the Ctrl and S.m samples. DPSC.7 cells expressed genes associated with the response to reactive oxygen species (ROS) response. DPSC.7 subgroup cells established characteristic aggregation under the stimulation of different pathogens in UMAP. The MAPK/ERK1/2 and NF-κB pathways were up-regulated, *DUSP1/5/6* expressions were suppressed, FOS synthesis was activated, the immune-related pathway was induced, and the levels of cytokines, including *IL-6* and *CCL2*, were up-regulated in DPSC.7 cells when stimulated with C.a.

**Conclusions:**

Our study analyzed the cellular and molecular properties of DPSCs infected by oral fungi and bacteria with single-cell RNA sequencing. A subcluster of DPSCs responded specifically to infections with different pathogens, activating the MAPK and NF-κB pathways to induce immune responses *via* the ROS pathway. This suggests novel treatment strategies for fungal infections.

## 1 Introduction

Microbial infections often cause organ dysfunctions and immune disorders threatening human health. Infectious diseases impose a substantial economic burden and constitute one of the most severe health issues worldwide (Barreto et al., 2006). Microbial pathogens colonize and invade many human organs, including the oral cavity. Various dental diseases are initiated by excess oral microflora. For instance, oral thrush and *Candida*-associated denture stomatitis are linked to fungal infection, dental caries, and periodontitis associated with bacterial irritation (Espinosa-Cristóbal et al., 2009; Farges et al., 2009; Gao et al., 2018; Xiao et al., 2018). Periodontal diseases, the putative pathogens of which include *Porphyromonas gingivalis* and *Tannerella forsythensis*, are highly prevalent and could affect up to 90% of the worldwide population (Pihlstrom et al., 2005; Patel et al., 2021).

*Candida albicans* and *Streptococcus mutans* are representative oral fungal and bacterial pathogens. *C. albicans* is the most common opportunistic fungal pathogen in the oral cavity. The carriage rates of *C. albicans* range from 20%-75% in general populations, 65%-88% in those living in acute and long-term care facilities, and 95% in those infected with human immunodeficiency virus (HIV) (Akpan and Morgan, 2002). Oral candidiasis is a common opportunistic infection caused by an overgrowth of *Candida* species in the oral cavity, the most frequent of which is *C. albicans* (Akpan and Morgan, 2002; Singh et al., 2014; Matsubara et al., 2017; Sherrington et al., 2017). *S. mutans* as one species of oral *streptococcus*, constitutes about 26.9% of all oral microorganisms (Costalonga and Herzberg, 2014) and is associated with dental caries (Hamada and Slade, 1980; Espinosa-Cristóbal et al., 2009; Pitts et al., 2017). Untreated, permanent teeth caries was the most prevalent health condition in 2019, affecting almost 2.0 billion prevalent cases worldwide (Wen et al., 2022).

Both *C. albicans* and *S. mutans* trigger inflammation of dental pulp cells. Under certain conditions, such as a compromised immune system or poor oral hygiene, *C. albicans* may overgrow and invade the pulp tissue. One meta-analysis found that *Candida* spp. account for 8.20% of root canal infections, approximately two-thirds of which were caused by *C. albicans* (Mergoni et al., 2018), underscoring the significant role that *C. albicans* plays in the etiology of endodontic diseases. The presence of this fungus may interfere with the normal functioning of pulp cells, leading to an inflammatory response and tissue damage. Caries-related pathogens may spread and irritate dental pulp cells through dentinal tubules (Hahn and Liewehr, 2007). *S. mutans* accounted for almost 8.1% of all *Streptococcus* in young permanent teeth with severe caries (Gross et al., 2010). The bacteria generate acidic byproducts that erode dental enamel, initiating cavity formation. As the cavity deepens, the invasive bacteria and their metabolites penetrate deeper into the tooth’s pulp, triggering inflammation and subsequent infection.

The dental pulp in the tooth center is mainly responsible for maintaining dentin and responding to diverse damages (Pagella et al., 2021; Lee et al., 2022). The dental pulp contains odontoblasts, fibroblasts, dental pulp stem cells, endothelial cells, and T cells (Vainio et al., 1993; Yin et al., 2021). Of these, the dental pulp stem cells (DPSCs) are clonogenic, highly proliferative, mesenchymal stem cells derived from human dental pulp (Gronthos et al., 2000; Miura et al., 2003). DSPCs are heterogeneous, not uniformly expressing many markers and reflecting differences in the developmental stages (Gronthos et al., 2000; Miura et al., 2003; Yamada et al., 2019; Yin et al., 2021). As an essential part of the innate immune response of dental pulp, DPSCs engage in tissue repair and secrete inflammatory cytokines to maintain homeostasis, and have immense potential for tissue regeneration (Liu et al., 2014; Cataldi et al., 2022).

Several issues require attention. Gronthos et al. considered DPSCs homogeneous cells and failed to capture the gene expression of each subcluster. It remains unclear how DPSC subclusters respond to different pathogens (Kawashima et al., 2005; Liu et al., 2014; Macosko et al., 2015; Mao et al., 2024). Also, although the effects of oral bacteria on DPSCs have been studied clearly, there is still the need to investigate the contribution of oral microbes from other species, including fungi and viruses, in dental caries and infected root canals. Furthermore, the specific mechanisms by which DPSCs subclusters respond to fungal infections remain largely unexplored, presenting an opportunity to uncover novel strategies for antifungal treatment.

Thus, we employed single-cell RNA sequencing (scRNA-seq) to capture the responses of DPSC subclusters infected by oral bacteria and fungi, represented by *S. mutans* and *C. albicans*, at single-cell resolution. We depict the DPSC transcriptome changes after infection, identify DPSC subclusters countering the fungal pathogen, and reveal the differentiation of DPSCs to suggest future, specific therapeutic approaches toward fungal infections.

## 2 Methods

### 2.1 DPSCs collection and cultivation

DPSCs were obtained from six healthy patients aged 18-23 years. All patients have informed written consent. The experimental protocol was approved by the Ethics Committee of Peking University School and Hospital of Stomatology (Approval No. PKUSSIRB-202163047). Immediately after tooth extraction, the pulp tissues were separated and minced into 0.1 × 0.1 × 0.1 cm^3^ cubes and digested with 3 mg/mL collagenase type I (Worthington). Then, the tissues were seeded into six-well plates containing minimal essential medium-α (α-MEM, Gibco) with 10% fetal bovine serum (FBS, Kang Yuan Biology) and 1% penicillin-streptomycin (Gibco), and incubated at 37°C under 5% CO_2_. Cells of passage 4 or 5 were applied (Zhang et al., 2023).

### 2.2 The *C. albicans* and *S. mutans* strains and the growth conditions

*Candida albicans* 90028 (*C. albicans*) and *Streptococcus mutans* UA159 (*S. mutans*) were obtained from the Central Laboratory of Peking University School of Stomatology. *C. albicans* was cultured in Sabouraud medium (5% CO_2_, 37°C). *S. mutans* was grown in brain heart infusion (BHI) medium supplemented with 1% hemin and 0.5% vitamin K in an anaerobic atmosphere.

### 2.3 Infection of DPSCs by *C. albicans* and *S. mutans*

All the DPSCs from six patients were mixed and seeded into 24-well plates at a density of 5 × 10^4^ per well and cultured overnight. The final concentration of the bacterial suspension was measured using the optical density at 630 nm (OD630). An OD_630_ = 0.1 was equivalent to a bacterial concentration of 1 × 10^9^ colony-forming units (CFU)/mL. The bacterial suspension was diluted to attain the desired multiplicity of infection (MOI). After DPSCs adhered to the plates, the cells were stimulated with *C. albicans* and *S. mutans* with a MOI of 1:1 for 4 h at 37°C with 5% CO_2_. There were no antibiotics in the post-infection medium.

### 2.4 scRNA-seq using the Singleron GECSCOPE platform

A hemocytometer and an inverted microscope were used to assess the quality and concentration of suspensions. We carried out single-cell preparations with 1 × 10^5^ cells/mL in concentration in PBS (HyClone) and loaded them onto microfluidic devices. scRNA-seq libraries were established by Singleron GEXSCOPE^®^ protocol utilizing GEXSCOPE^®^ Single-Cell RNA Library Kit (Singleron Biotechnologies). Then, individual libraries were diluted to 4 nM, pooled, and subjected to paired-end sequencing on the Illumina Novaseq 6000 platform.

### 2.5 scRNA-seq alignment and unique molecular identifier calculation

First, raw reads were processed using the CeleScope pipeline (https://github.com/singleron-RD/CeleScope, version 1.9.0) to remove low-quality reads and Cutadapt v1.17 to trim poly-A tail and adapter sequences (Martin, 2011). We extracted the cell barcodes and the UMIs. Then, STAR (version 2.6.1a) was applied to map reads to GRCh38 (ensembl version 92 annotation) (Dobin et al., 2013). UMI counts and gene counts were obtained with featureCounts (version 2.0.1) and used to produce expression matrix files for future analysis (Liao et al., 2014).

### 2.6 Data importation, transformation, and integration

We imported the database derived as described above into Origin Software (2020b) and R software (version 4.0.3). The filtered, feature barcode matrices were inputted using the ‘Read10X’ and ‘CreateSeuratObject’ functions. The Seurat R package (version 4.0.2) was applied to control quality, normalize the database, and facilitate downstream analysis (Stuart et al., 2019). Cells expressing 200 to 4,000 genes and less than 15% mitochondrial genes were reserved to exclude low-quality and artifact cells. We normalized data to transform gene expression matrices. Analysis of integrated data and the correct batch was performed with the ‘FindIntegrationAnchors’ and ‘IntegrateData’ functions.

### 2.7 Dimensionality reduction, cell clustering, and identification

We used ‘CellCycleScoring’ and ‘ScaleData’ to mitigate the influence of cell cycle heterogeneity (Tirosh et al., 2016). Then, the ‘FindNeighbors’ and ‘FindClusters’ functions (reduction.type = pca, resolution = 1.0, dims = 1:50) were employed to define cell clusters. The ‘FindAllMarkers’ function was used to identify specific marker genes of each cluster.

### 2.8 Gene ontology categories and gene set variation analysis

Significantly upregulated cluster-defining genes were computed and carried out gene set over-representation with the gsfisher R package (https://github.com/sansomlab/gsfisher, version 0.2) and acquired gene sets from the GO database.

GSVA, a non-parametric and unsupervised analytical method, was employed to evaluate the relative regulation of pathway activity by comparing different samples using the GSVA package (version 1.38.2). Pathway gene sets were downloaded from the GSEA database (https://www.gsea-msigdb.org/gsea/index.jsp, h.all.v7.5.1.symbols and c5.all.v7.5.1.symbols) (Subramanian et al., 2005). Also, we applied the R package AUCell (version 1.12.0) to calculate the area under the curve (AUC) scores of each cell for pathway activities with default parameters.

### 2.9 Transcription factor module analysis

The R package SCENIC workflow (https://github.com/aertslab/SCENIC, version 1.2.4, RcisTarget version 1.10.0, and AUCell version 1.12.0) was used to analyze active TF modules in DPSC.7 subgroups (Aibar et al., 2017). We inputted the raw UMI counts for each sample in SCENIC. Then, we applied the GENIE3 method to identify potential TF targets according to the standard SCENIC procedure. AUCell was used to compute a score for each TF module of all cells.

### 2.10 Pseudotime ordering and lineage trajectories

The pseudotime trajectories were identified using the Monocle2 (version 2.18.0) R package to discover potential developmental transitions (Qiu et al., 2017; Cao et al., 2019). 10,000 cells were selected from the database randomly and imported into Monocle2. Then, we followed the standard workflow and preprocessing steps.

## 3 Results

### 3.1 A DPSCs subcluster proportion, DPSC.7, displaying a specific characteristic

**Figure 1:**
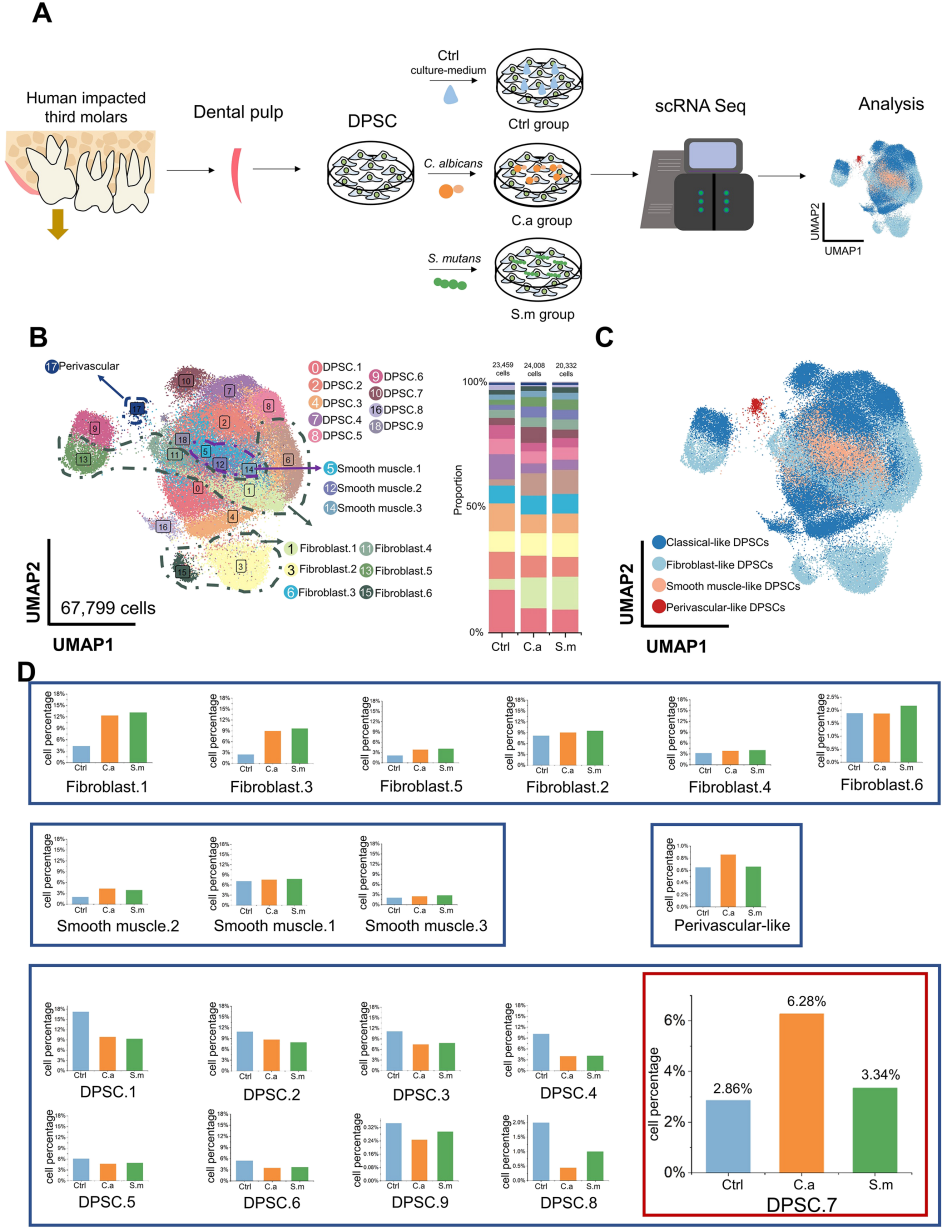
Specific characteristics of DPSC.7 infected by *C. albicans* and *S. mutans*. **(A)** Schematic representation of scRNA-seq experimental design strategy. **(B)** Distribution of 19 subclusters of DPSCs visualized in UMAP (left) and bar graph of relative cell proportion divided by samples (right). **(C)** UMAP representation of 4 major cell types, including classical-like DPSCs, fibroblast-like DPSCs, smooth muscle-like DPSCs, and perivascular-like DPSCs. **(D)** Bar graphs showing the cell percentage of 19 subclusters of DPSCs. DPSC.7 percentage in the C.a sample was approximately twice in the Ctrl and the S.m samples.

DPSCs were collected from the impacted third molars of six healthy subjects. Then, we mixed DPSCs and separated them into three samples, one of which was cultured with cell medium without additional microbial stimulation (Ctrl), and two of which were infected with *Candida albicans* (C.a) and *Streptococcus mutans* (S.m), respectively, to emulate the early stages of microbial infection (Infected). We performed scRNA-seq to elucidate the cellular and molecular characteristics of DPSCs (**Figure 1A**).

After quality control, 67,799 cells were yielded in total, including 23,459 cells in the Ctrl sample, 24,008 cells in the C.a sample, and 20,332 cells in the S.m sample (**Supplementary Figure S1**). Nineteen subclusters were identified through unsupervised clustering and further annotated (**Figure 1B**; **Supplementary Figures S2**, **S3**). Since several subclusters shared common cellular and functional properties, we integrated cell subclusters, which resulted in four major clusters, including classical-like DPSCs, fibroblastic-like DPSCs, smooth muscle-like DPSCs, and perivascular-like DPSCs (**Figure 1C**; **Supplementary Table S1**).

We observed the change in cell percentage under the different conditions of infection. The proportion of several subclusters increased in both C.a and S.m samples, including Fibroblast.1, Fibroblast.3, Fibroblast.5, and Smooth muscle.2, which expressed genes involved in responding to microbial infection, such as toll-like receptor signaling pathway, regulation of chemotaxis, and ossification; these are the common pathway for resistance to pathogens, consistent with previous studies (**Supplementary Figure S4**) (Kawashima et al., 2005; Liu et al., 2014; Cataldi et al., 2022).

No apparent differences in cell ratio in a few subclusters were shown between the Infected and Ctrl samples, including Fibroblast.2, Fibroblast.4, Fibroblast.6, Smooth muscle.1, and Smooth muscle.3. Also, the percentage of six subclusters in the Infected samples was lower than that in the Ctrl sample (DPSC.1, DPSC.2, DPSC.3, DPSC.4, DPSC.5, and DPSC.6), expressing a gene signature consistent with cell cycle-related pathways. Interestingly, a subcluster, DPSC.7, showed a specific characteristic. Cell proportion in the C.a sample was more than twice higher than that in the Ctrl sample and approximately twice higher than that in the S.m sample (Ctrl [672 cells, 2.86%], C.a [1507 cells, 6.28%], S.m [680 cells, 3.34%], **Figure 1D**).

### 3.2 Characteristic aggregation shown in DPSC.7 under different pathogens stimulation

**Figure 2:**
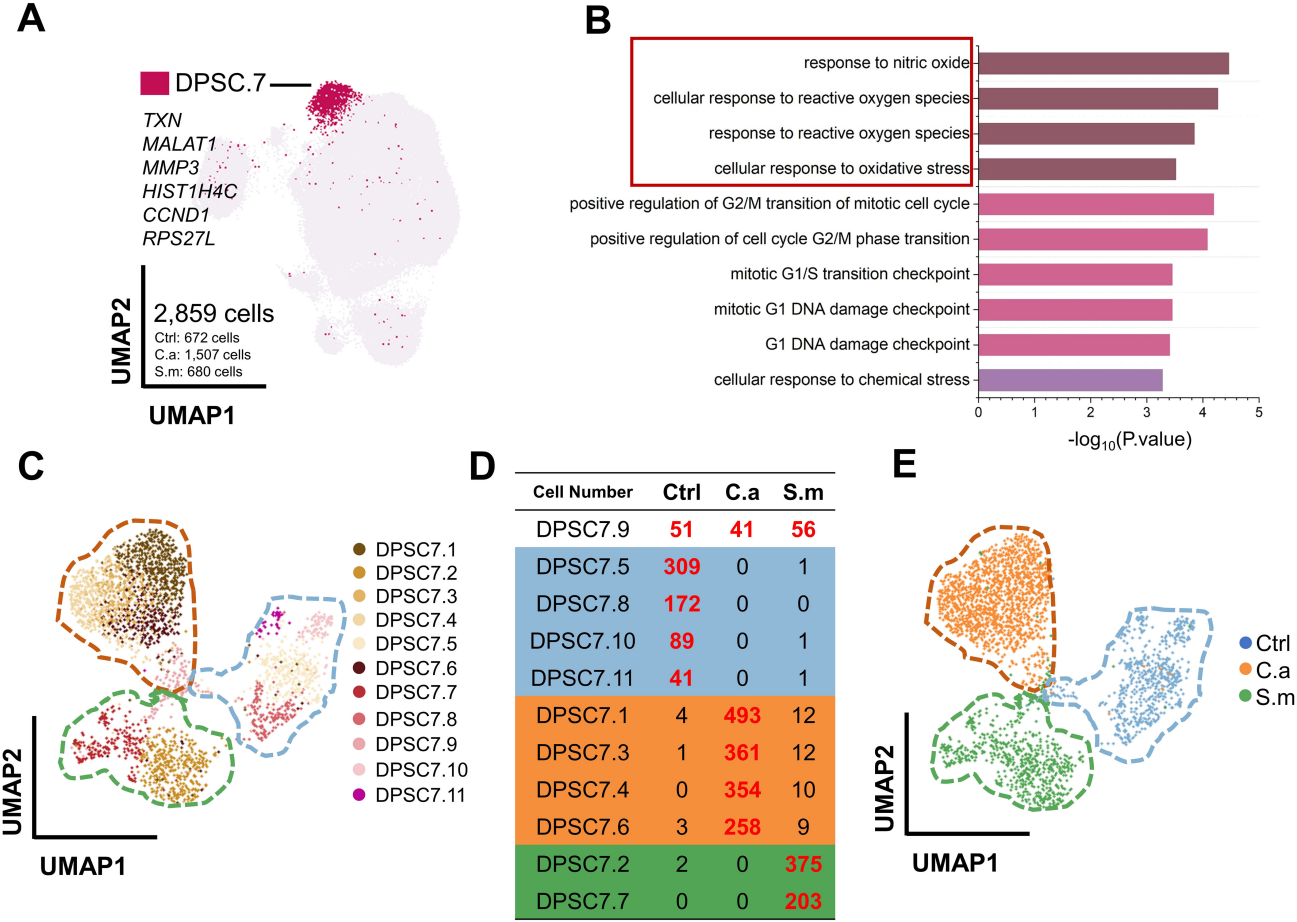
DPSC.7 established specific aggregation under *C*. *albicans* and *S. mutans* infection. **(A)** UMAP plot showing DPSC.7 cells (2,859 cells, Ctrl [672 cells], C.a [1,507 cells], S.m [680 cells]), whose marker genes were *TXN*, *MALAT1*, *MMP3*, *HIST1H4C*, *CCND1*, and *RPS27L*. **(B)** Bar graphs depicting GO terms enriched in DPSC.7. **(C)** Eleven DPSC.7 subclusters were identified and divided by unsupervised clustering shown in UMAP. **(D)** List showing cell number of eleven DPSC.7 subclusters, which were divided into four categories, including Class One distributed in three samples (DPSC7.9), Class Two primarily from cells in Ctrl sample (DPSC7.5, DPSC7.8, DPSC7.10, and DPSC7.11), Class Three mainly composed of cells in C.a sample (DPSC7.1, DPSC7.3, DPSC7.4, and DPSC7.6), and Class Four mainly consisting of cells in S.m sample (DPSC7.2 and DPSC7.7). **(E)** UMAP plot visualization of DPSC.7 separated by Ctrl, C.a, and S.m samples distinctly, where cells in the Ctrl sample were distributed on the left side of the plot, cells in the C.a sample were dispersed on the upper right side of the plot, and cells in the S.m sample were occupied in the lower right side of the plot.

DPSC.7 expressed signature genes, including *TXN*, *MALAT1*, *MMP3*, *HIST1H4C*, *CCND1*, and *RPS27L*, linked to response to reactive oxygen species (ROS), chemical stress, and cell cycle-related pathway (**Figures 2A, B**; **Supplementary Tables S2**, **S3**). To determine whether there were specific subgroups in DPSC. 7 responding to *C. albicans* infection, we carried out further subgroup clustering.

This clustering resulted in eleven DPSC.7 subgroups (**Figure 2C**; **Supplementary Table S4**). Surprisingly, these subgroups were further divided into four categories (**Figure 2D**). Cells of the Class One subgroup existed in three samples (DPSC7.9), distributed in the junction center of three samples in a UMAP plot. In subgroups of Class Two, most cells were in the Ctrl sample (DPSC7.5, DPSC7.8, DPSC7.10, and DPSC7.11) occupied on the right side of the UMAP plot. In subgroups of Class Three, cells were mainly in the C.a sample (DPSC7.1, DPSC7.3, DPSC7.4, and DPSC7.6), displayed in the upper left of the UMAP plot. In subgroups of Class Four, most of the cells were in the S.m sample (DPSC.2 and DPSC7.7), exhibiting in the lower left of the UMAP plot. DPSC.7 subgroups exhibited characteristic aggregation in response to stimulation by different pathogens (**Figure 2E**).

### 3.3 DPSC.7 subgroups infected by *C. albicans* expressing genes involved in ERK1/2 signaling pathway

**Figure 3:**
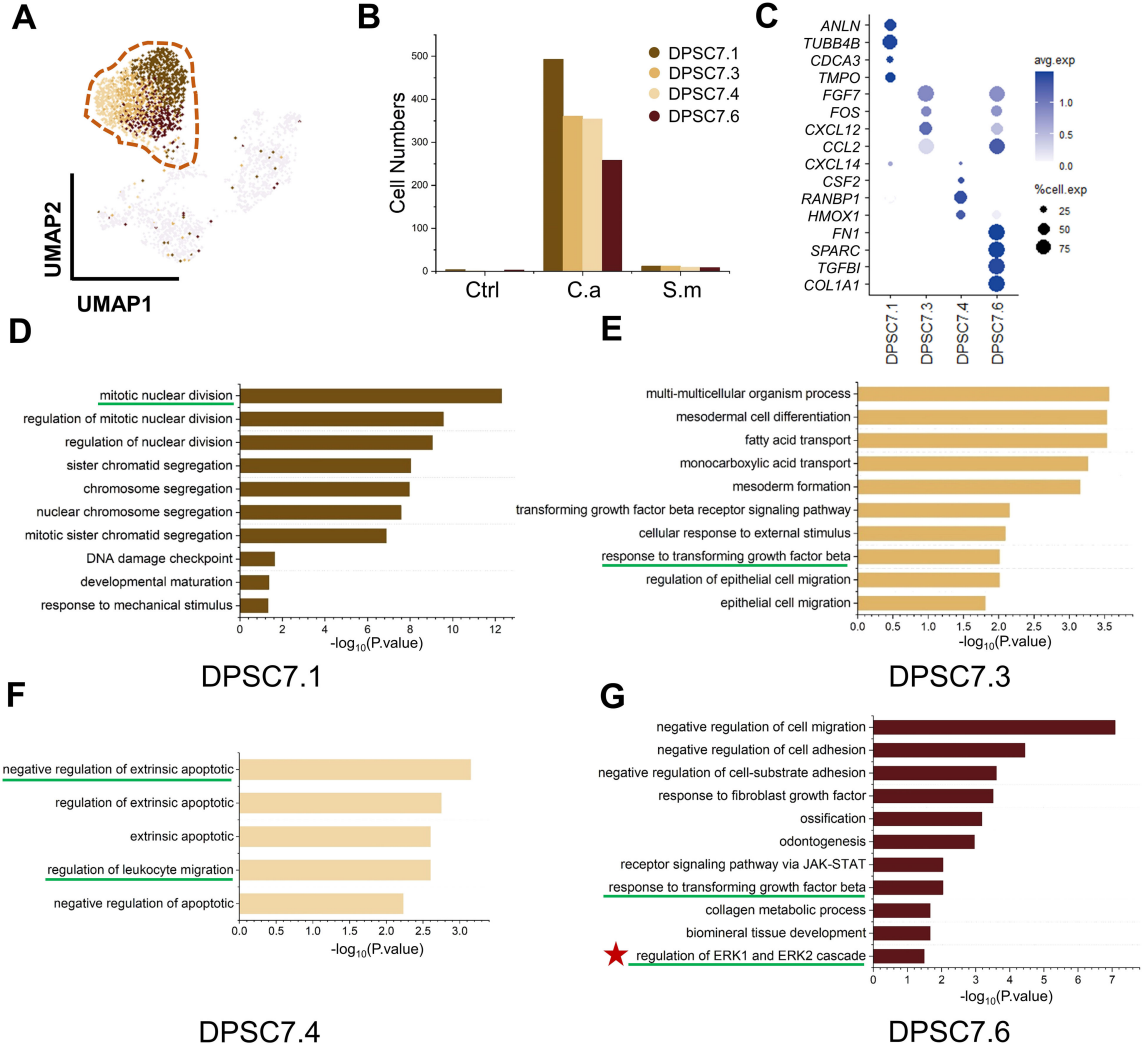
DPSC.7 subgroups cells in C.a sample expressed gene related to ERK1/2 pathway. **(A)** UMAP plot of DPSC7.1, DPSC7.3, DPSC7.4, and DPSC7.6. **(B)** Bar graphs showing the cell number of DPSC7.1 (Ctrl [4 cells], C.a [493 cells], and S.m [12 cells]), DPSC7.3 (Ctrl [1 cell], C.a [361 cells], and S.m [12 cells]), DPSC7.4 (C.a [354 cells], and S.m [10 cells]) and DPSC7.6 (Ctrl [3 cells], C.a [258 cells], and S.m [9 cells]) in three groups. **(C)** Dot plots showing the expression of signature genes and the percentage of cells expressing each gene for four subgroups, including DPSC7.1, DPSC7.3, DPSC7.4, and DPSC7.6. Expression values were normalized and scaled averages. **(D–G)** Bar graphs showing pathways enriched in DPSC7.1 **(D)**, DPSC7.3 **(E)**, DPSC7.4 **(F)**, and DPSC7.6 **(G)**.

To discover how DPSC.7 might respond to *C. albicans* infection, we performed differentially expressed genes (DEG) and pathway enrichment analysis on eleven subgroup cells of DPSC.7 (**Figures 3A–C**; **Supplementary Figures S5**, **S7**).

Four subgroups mainly existed in the C.a sample (**Supplementary Table S5**). DPSC7.1 subgroup cells were linked to the cell-cycle pathway (**Figure 3D**). DPSC7.3 subgroup cells characterized by the expression of *FGF7*, *FOS*, *CXCL12*, and *CCL2* were associated with the TGF-β receptor signaling pathway and epithelial cell migration (**Figure 3E**). DPSC7.4 subgroup cells displayed gene signatures related to negative regulation of extrinsic apoptotic and regulation of leukocyte migration (*CXCL14*, *CSF2*, *RANBP1*, *HMOX1*) (**Figure 3F**). DPSC7.6 subgroup cells expressed genes involved in response to TGF-β, collagen metabolic process, and regulation of ERK1 and ERK2 cascade (**Figure 3G**). Marker genes and enrichment pathways in other subgroups are described in **Supplementary Figures S6**, **S7** (**Supplementary Tables S10**–**S13**).

### 3.4 Activation of MAPK/P38, MAPK/ERK1/2 signaling pathways in DPSC.7 subgroups

**Figure 4:**
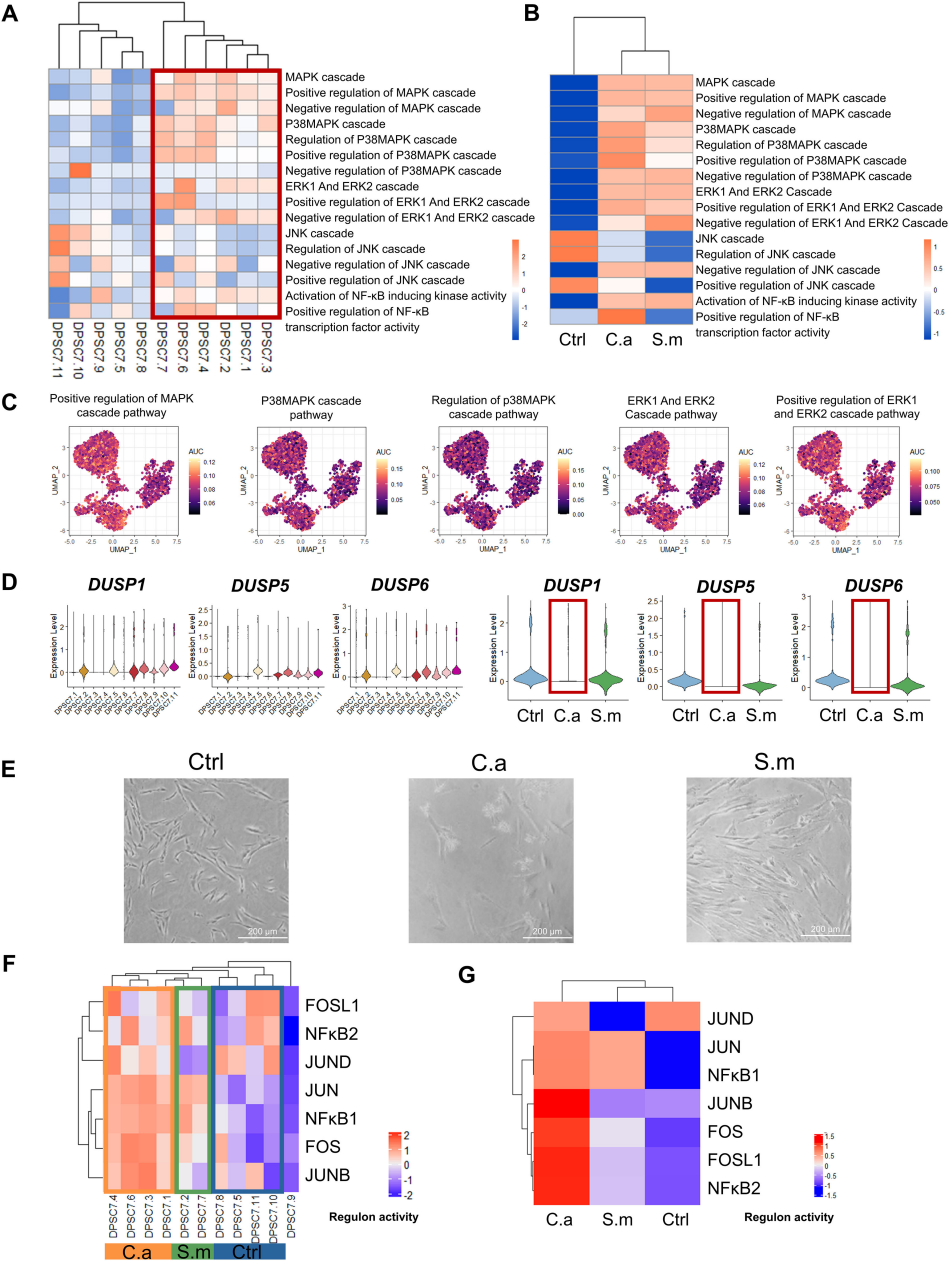
The activity of pathways in DPSC.7 subgroups cells infected by *C*. *albicans*. The activity of MAPK/P38, MAPK/ERK1/2 signaling pathways, and transcription factors, including FOS and FOSL1, was up-regulated, and expression of *DUSP1/5/6* decreased in the DPSC.7 subgroup under *C.albicans* infection. **(A, B)** Heatmap showing the differences of pathway activity associated with MAPK and NF-κB signaling pathway by GSVA enrichment scores among eleven DPSC.7 clusters **(A)** and three samples **(B)**. **(C)** UMAP of AUC scores of pathways related to MAPK signaling pathway in DPSC.7. **(D)** Violin plots of *DUSP1*, *DUSP5*, and *DUSP6* expression among eleven DPSC.7 clusters and three samples. **(E)** Imaging of DPSCs in Ctrl, C.a, and S.m samples under the microscope (Scale = 200 μm). **(F, G)** Heatmap showing the scaled regulon activity by the subclusters of DPSC.7 **(F)** and three samples **(G)**.

Our results showed that DPSC7.6 expressed genes linked to ERK1 and ERK2 pathways (**Figure 3G**). Previous studies have described human cells’ response to *C. albicans via* NF-κB and a biphasic MAPK pathway. Activation of NF-κB and the first MAPK phase – c-Jun might be related to fungal cell wall recognition. And activation of the second MAPK phase – MKP1 and c-Fos, relying on hypha formation and fungal burdens, was associated with proinflammatory responses (Moyes et al., 2010; Naglik et al., 2014; Böhringer et al., 2016; Rast et al., 2016). Therefore, we used GSVA and AUcell to calculate the activity of MAPK and related pathways in DPSC.7 subgroups. We found the activity of MAPK, P38, ERK1/2, and NF-κB pathway in the Infected group was higher than that in the Ctrl group, but there was no apparent difference between C.a and S.m groups (**Figures 4A–C**).

MKP1(DUSP1) and c-FOS activation are related to response specifically to damage-inducing hyphae (Moyes et al., 2010). Thus, we detected the expression of dual specificity phosphatase (DUSP) family genes. DUSP is the key phosphatase acting as the negative regulator of MAPK activity, which could recognize and inactivate ERK1/2, P38, and JNK (Owens and Keyse, 2007; Keyse, 2008). Of interest, the expression of *DUSP1/5/6* in the C.a sample was lower than that in the Ctrl and S.m sample (**Figure 4D**). However, it was not the result of the lack of existence of hyphae. Our microscopic results showed that *C. albicans* hypha had formed and DPSCs were in contact with hyphae before sequencing (**Figure 4E**). Besides, our TF activity analysis demonstrated that the activity level of FOS in the C.a sample, other key factors response to *C. albicans*, was higher than that in the Ctrl and S.m sample with SCENIC. At the same time, the activities of FOS, FOSL1, and JUNB in the C.a group were higher than those in Ctrl and S.m samples (**Figures 4F, G**; **Supplementary Figure S8**, **Supplementary Table S6**).

### 3.5 The same trend of MAPK/ERK1/2 and ROS pathway activity with the pseudotime

DPSC.7 displayed a gene signature corresponding to the ROS pathway (**Figure 2B**). The activity of the ROS pathway in the C.a sample was higher than that in the other two samples. Previous studies indicated that NF-kB and MAPK have been the best-characterized oxidation-reduction-sensitive pathways (Baas and Berk, 1995; Ushio-Fukai et al., 1998; Sano et al., 2001; Fukawa et al., 2012). Moreover, MAP kinase phosphatase has been sensitive to ROS, and ROS promotes sustained MAPK pathway activation (Kamata et al., 2005; Levinthal and Defranco, 2005; Cagnol and Chambard, 2010).

**Figure 5:**
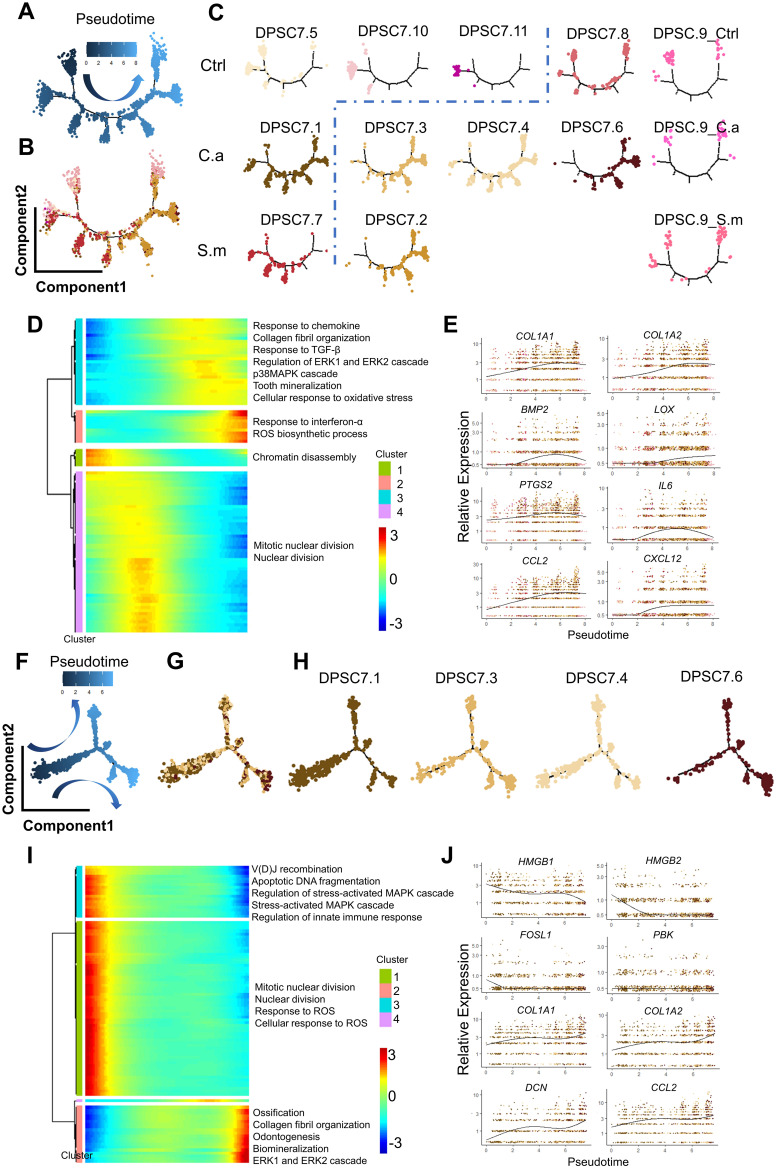
Pseudotime trajectory of DPSC.7. The pseudotime trajectory of DPSC.7 suggests that the activity of MAPK/ERK1/2 and immune-related pathways increased with the upregulation of the ROS pathway. **(A–C)** Monocle 2 trajectory plot showing the dynamics of DPSC.7 and the pseudotime curve **(A)** and DPSC.7 subclusters together **(B)** and respectively **(C)**. **(D)** The differentially expressed genes were hierarchically clustered into four subclusters along with the pseudotime of DPSC.7. The annotated GO terms of each cluster were selected and provided. **(E)** Specific genes associated with pathways chosen in **(D)**, including *COL1A1*, *COL1A2*, *BMP2*, *LOX*, *PTGS2*, *IL6*, *CCL2*, and *CXCL12*, are listed to describe expression kinetics. **(F–H)**. Pseudotime trajectory analysis shows the relationships of nine subclusters of mesenchymal cells, colored by pseudotime **(F)** and by major clusters together **(G)** and separately **(H)**. **(I)** The differentially expressed genes were hierarchically divided into four subgroups through pseudotime in DPSC7.1, DPSC7.3, DPSC7.4, and DPSC7.6. The annotated GO terms of each cluster were selected and provided. **(J)** Several genes related to pathways chosen in **(I)**, including *HMGB1*, *HMGB2, FOSL1, PBK, COL1A1*, *COL1A2*, *DCN*, and *CCL2*, are listed to show expression kinetics.

Therefore, we used Monocle2 to explore the relationship between ROS and MAPK pathways. We found that cells in several subgroups, including DPSC7.1, DPSC7.5, DPSC7.7, DPSC7.10, and DPSC7.11, were mainly distributed in the early phase of pseudotime, associated with expressed genes related to cell cycle regulation. Cells in other subgroups were mostly in the late phase of pseudotime, related to expressed genes involved in immune response and mineralization (**Figures 5A–C**). Along the pseudotime, the activity of the ROS biosynthetic process was raised, accompanied by the increased activity of MAPK/ERK1/2 and immune-related pathways, whose related genes were *COL1A1*, *LOX*, *IL6*, and *CXCL12* (**Figures 5D, E**; **Supplementary Table S7**). Besides, we constructed pseudotime analysis in DPSC7.1, 7.3, 7.4, and 7.6. The activity of ROS and MAPK pathways was the highest at the early phase of pseudotime. Along the pseudotime, the activity of ERK1/2 and immune-related pathways was up-regulated, whose related genes were *HMGB1*, *FOSL1, DCN*, and *CCL2* (**Figures 5F–J**; **Supplementary Table S8**).

### 3.6 Activation of immune-related pathway and cytokine expression in DPSC.7 subgroups

**Figure 6:**
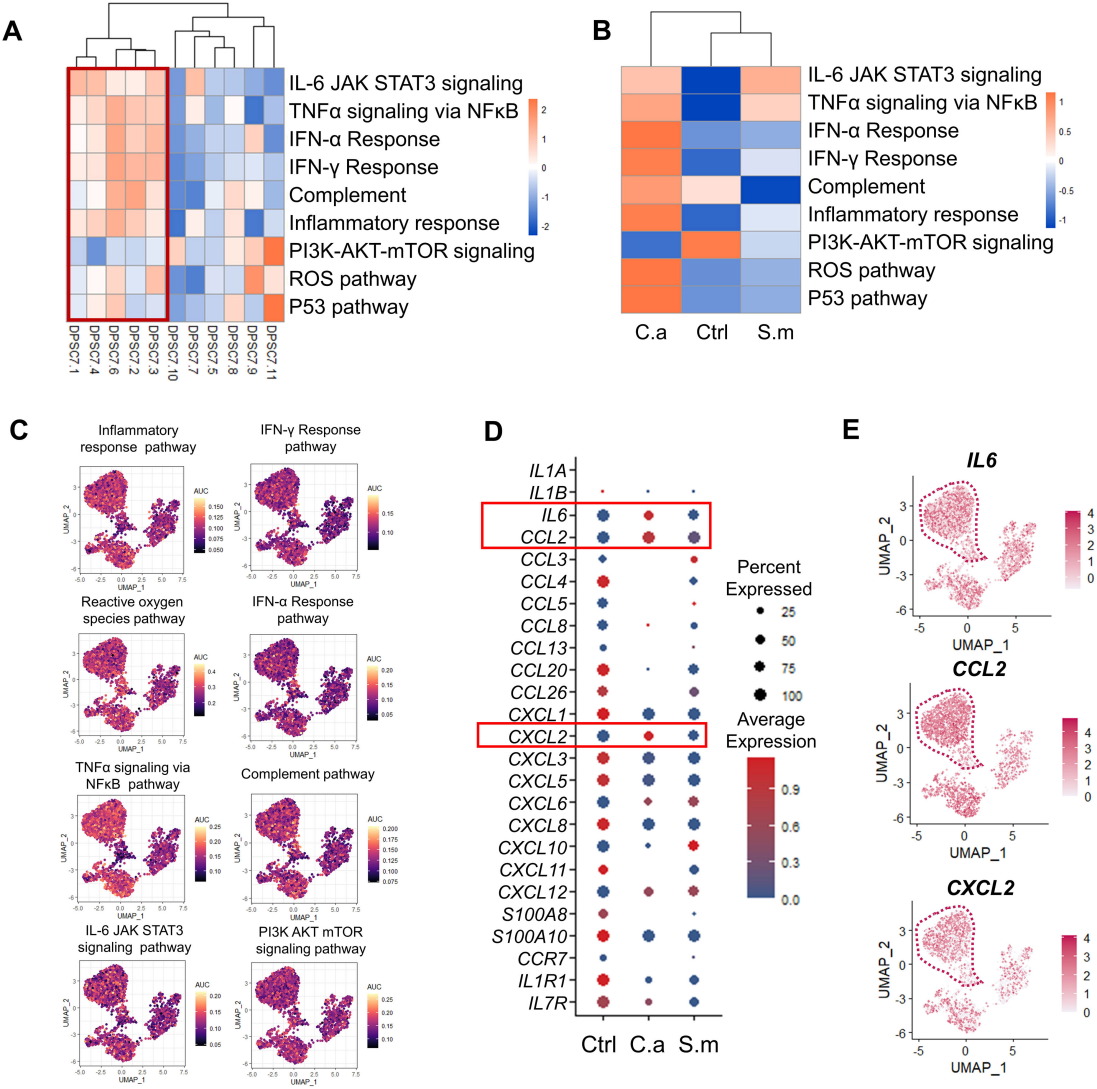
The activity of immune-related pathways and genes was up-regulated in the C.a sample. **(A, B)** Heatmap representing the GSVA enrichment score for the pathway related to inflammation-related responses among 11 subclusters **(A)** and three samples **(B)**. **(C)** UMAP visualization of individual cell AUC score for the selected immune-related pathway. **(D)** Dot plot indicating chemokine receptor and chemokine expression in DPSC.7 among three samples, whose expression values were normalized and scaled averages. **(E)** UMAP plots reveal the expression of *IL6*, *CCL2*, and *CXCL2* in each cell of DPSC.7.

Finally, we detected the activity of the immune-related pathway in each subgroup. The activity of the immune-related pathway in the C.a sample, including inflammatory response, complement, and IFN-α response, was higher than that in the Ctrl and the S.m samples (**Figures 6A–C**). In addition, the average expression of *IL-6*, *CCL2*, and *CXCL2* and the percentage of *IL-6*, *CCL2*, and *CXCL2* positive cells in the C.a group were also higher than that in the Ctrl and the S.m sample (**Figures 6D, E**; **Supplementary Figure S9**).

At the same time, we observed that the activity of TGF-β/SMAD pathways increased in the Infected sample. Transforming growth factor–β (TGF-β) was reported to induce collagen deposit around newly recruited polymorphonuclear cells to avoid microbial diffusing (Santus et al., 2017). Similarly, we also found that the activity of odontogenic, ossification, and biomineralization pathways in the Infected sample was higher than in the Ctrl sample (**Supplementary Figure S9**, **Supplementary Table S9**).

## 4 Discussion

Our study analyzed the cellular and molecular characteristics of DPSCs stimulated by oral fungi and bacteria infections represented by *Candida albicans* and *Streptococcus mutans* by scRNA-seq. DPSCs were divided into four major clusters: classical-like DPSCs, fibroblastic-like DPSCs, smooth muscle-like DPSCs, and perivascular-like DPSCs. These clusters exhibited both convergent and divergent responses to the various pathogenic stimuli. We found a specific subcluster, DPSC.7, expressed genes linked to the ROS pathway. DPSC.7 subgroups showed characteristic aggregation under different pathogen infections. The activity of MAPK/P38, MAPK/ERK1/2, and NF-κB signaling pathways were up-regulated, transcription factors (FOS, FOSL1, JUN, JUNB) were activated, *DUSP1/5/6* expression decreased, and immune-related pathway and cytokine expression were driven in DPSC.7 subgroups infected by *C. albicans*. Also, the activity of MAPK/ERK1/2 and ROS pathways had the same trend with pseudotime analysis. This finding might be instrumental in searching for new treatment strategies for antifungal infections.

In our study, the activity of MAPK/P38, MAPK/ERK1/2, and NF-κB pathway of DPSC.7 in the Infected sample was higher than that in the Ctrl sample. Our findings corroborate the established knowledge that *C. albicans* can elicit NF-κB and biphasic MAPK signaling pathways across diverse cell types, leading to the activation of immune-related pathways (Moyes et al., 2010, Moyes et al., 2011, Moyes et al., 2014; Naglik et al., 2014; Böhringer et al., 2016; Nikou et al., 2022). In particular, oral epithelial cells could orchestrate an innate response against *C. albicans* infection through NF-κB and biphasic MAPK signaling pathways. Activation of NF-κB, the first MAPK phase, and transcription factor c-Jun was independent of morphology due to the recognition of fungal cell walls. The second MAPK phase was likely associated with *C. albicans* hyphal invasion and induction of inflammatory mediators (Moyes et al., 2010, Moyes et al., 2011).

Moyes et al. have identified two pivotal factors in the secondary mitogen-activated protein kinase (MAPK) phase: MKP-1 and c-Fos. These factors potentially form a danger response mechanism, enabling cells to remain quiescent in the presence of “commensal” fungal levels, while eliciting a specific and robust host response to “dangerous” fungal burdens (Moyes et al., 2010, Moyes et al., 2011). In our study, we noted an upregulation of FOS and a down-regulation of DUSP1/5/6 in the C.a group compared to the Ctrl and S.m samples, corroborating previous findings that c-Fos activation is strongly associated with mycelial growth and fungal burden (Moyes et al., 2010, Moyes et al., 2011). Microscopic imaging further substantiated the hyphae formation and the intimate contact between DPSCs and hyphae (**Figure 4E**). The c-Fos pathway was also found to be closely associated with the induction of inflammatory mediators, while DUSP1 activation ensured a potent yet tightly regulated immune response of DPSCs to *C. albicans* hyphae (Moyes et al., 2010, Moyes et al., 2011). Our findings lead us to hypothesize that DPSC.7 infected by *C. albicans* mycelia may partially activate immune-related cellular functions rather than informing or protecting. Prior research has indicated that the yeast form of *C. albicans* triggers c-Jun but fails to initiate a sufficient immune response, whereas its mycelial form activates c-Fos, prompting a vigorous immune reaction in oral epithelial cells (Moyes et al., 2010, Moyes et al., 2011; Naglik et al., 2014). Our study extends this by demonstrating that DPSC.7 infected by *C. albicans* hyphae activates FOS and immune-related pathways, in contrast to the stimulation by *S. mutans*, which induces JUN and a less robust immune response, aligning with previous studies (Moyes et al., 2010; Warnatsch et al., 2017).

Then, we explored why DUSP was suppressed in DPSC.7 in the C.a sample. Interestingly, we found that the activity of the ROS pathway was up-regulated in the C.a sample. DUSP was sensitive to ROS, and ROS could further prolong the activity of MAPK-related pathways by inhibiting DUSP (Kamata et al., 2005; Levinthal and Defranco, 2005; Cagnol and Chambard, 2010). These results suggested that DPSC.7 infected by *C. albicans via* ROS pathway inhibits *DUSP* expression and activates MAPK/ERK1/2 and MAPK/p38 pathway to provoke an immune-related response. However, although ROS might drive the activation of immune-related responses, the decrease of *DUSP* expression might lead to deleterious MAPK-related inflammatory responses or even apoptosis (Kamata et al., 2005; Cagnol and Chambard, 2010; Moyes et al., 2010).

ROS might also have other roles during the process of *C. albicans* infection. NF-κB and MAPK were the typical ROS-sensitive signaling pathways, and the increase of ROS would lead to the activation of MAPK and NF-kB pathways (Baas and Berk, 1995; Ushio-Fukai et al., 1998; Sano et al., 2001; Fukawa et al., 2012). ROS could drive inflammatory and immune responses (Hervera et al., 2018; Liu et al., 2022). Additionally, Warnatsch et al. revealed ROS’s function as microbe size sensors. Tiny microbes, such as *C. albicans* yeast, triggered ROS intracellularly and selectively suppressed IL-1β expression to restrict neutrophil recruitment. Giant microbes, such as *C. albicans* hypha, impelled ROS extracellularly and enlarged IL-1β expression to recruit a large number of neutrophils (Warnatsch et al., 2017). In our study, the size of *S. mutans* was relatively smaller, and ROS and immune-related pathways were not obviously activated; in comparison, the size of *C. albicans* hypha was larger, and ROS and immune-related pathways were provoked. Thus, a possible mechanism and hypothesis might be that ROS, as sensors of microbe size, amplified immune-related pathway activity by *C. albicans* hyphae and selectively inhibited the expression of cytokines by *S. mutans* in DPSCs, which was an essential part of the innate immune response.

We also found that the activity of TGF/SMAD was up-regulated in DPSC.7 infected by *C. albicans* and *S. mutans*, as well as activation of the fibrosis and mineralization pathways. TGF-β was recognized as a well-known profibrotic factor (Meng et al., 2016). Also, it was demonstrated that the fibrosis process emerged after pathogen infection in various kinds of cells (Meng et al., 2016; Chen et al., 2019; Lai et al., 2022). Santus et al. described the possible mechanism of TGF-β in *C. albicans* skin infections. Two major phases of the innate response to *C. albicans* infections included protective containment (abscess) and elimination (expulsion) phases. TGF-β could lead to collagen deposition and impede microbial diffusion to minimize tissue damage and optimize pathogen elimination during the early containment phases (Santus et al., 2017). Besides, the TGF-β/SMAD pathway was also related to tooth regeneration and biomineralization to help resist microbial irritation (Kaufman and Skrtic, 2016; Widbiller et al., 2018; Hu et al., 2019). Our study showed similar results. TGF-β/SMAD and related pathways, including collagen metabolic process and biomineralization, were activated in DPSC.7 infected by microflora. Moreover, *MALAT1*, a marker gene of DPSC.7, was demonstrated to regulate various factors, such as TGFβ-1 and BMP2, participating in the mineralization of dental pulp cells (Li et al., 2022). Besides, ROS were indicated to modulate TGF-β signaling through different pathways. ROS would activate TGF-β1 and regulate the fibrogenic effect of TGF-β (Jain et al., 2013; Beshay et al., 2020). Thus, TGF-β might be involved in limiting DPSC.7 migration to control microbial damage, and ROS might promote the TGF-β pathway.

**Figure 7:**
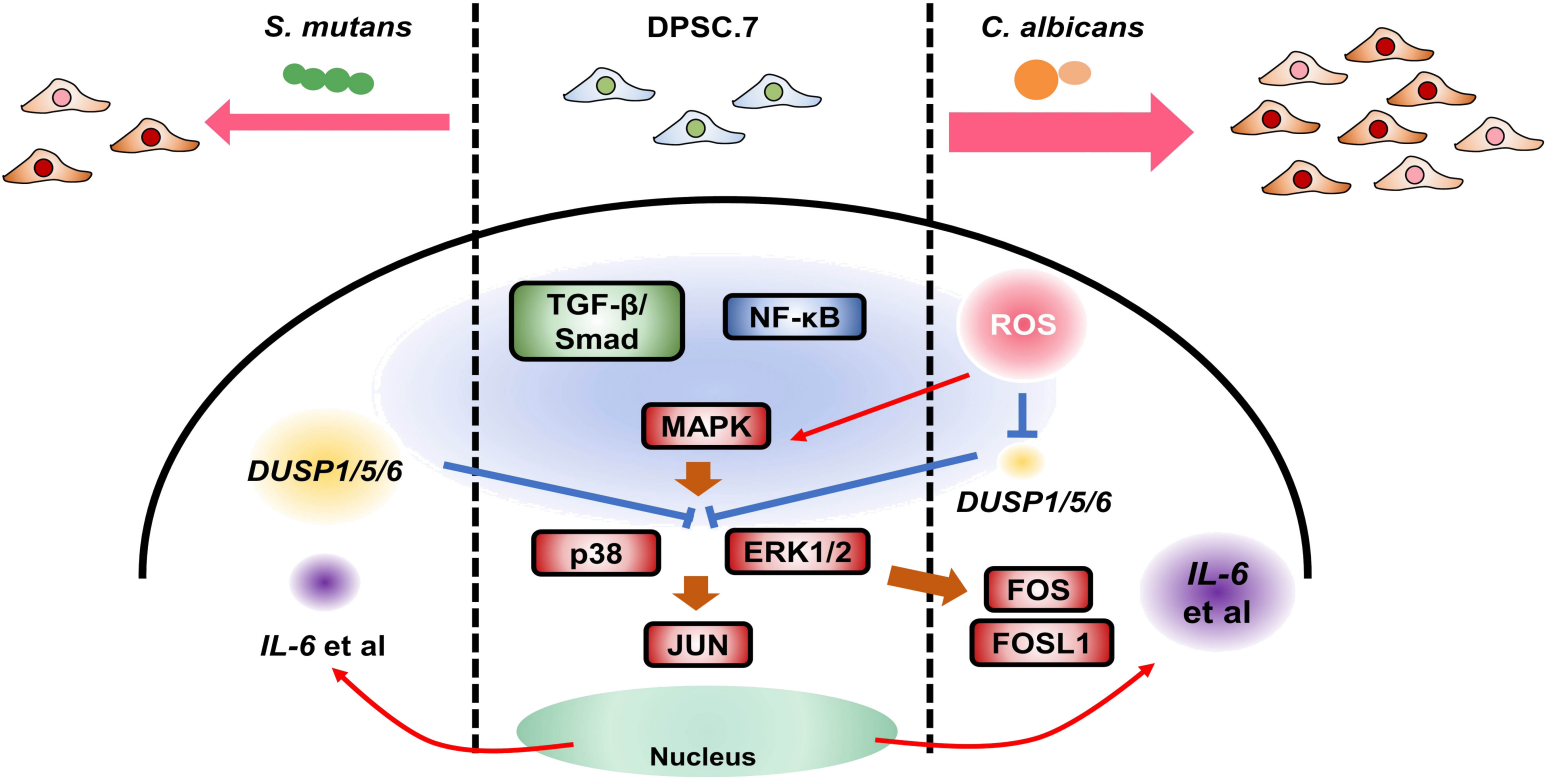
The possible mechanism model responding to oral pathogen infection in DPSC.7. *Candida albicans*, an oral opportunistic fungus, activated MAPK/p38, ERK1/2, and NF-κB pathway through ROS pathway, suppressed *DUSP1/5/6* expression, up-regulated transcription factors (FOS, FOSL1), drove immune response and increased cytokines expression (*IL6*, *CCL2*). Meanwhile, the activation of the TGF-β/SMAD signaling pathway induced the expression of genes related to fibrosis and mineralization to limit microbial diffusing. Also, *Streptococcus mutans*, an oral pathogenic bacterium, could trigger MAPK/P38, ERK1/2, NF-κB, and TGF-β/SMAD pathway, but activated JUN, to induce and weaken immune response and fibrosis response. This study provided new possibilities for antifungal infection with DPSCs in the dental pulp and other kinds of human diseases.

Therefore, we propose a potential mechanism model illustrating the response of DPSC.7 to oral pathogen infection. *C. albicans* initiates a cascade *via* the ROS pathway, activating the MAPK/p38, MAPK/ERK1/2, and NF-κB pathways, coupled with the suppression of *DUSP1/5/6* expression and the induction of key transcription factors. It also propels an immune response, characterized by an upregulation in the expression of cytokines, including *IL6* and *CCL2*. Meanwhile, the engagement of the TGF-β/SMAD signaling pathway stimulates the expression of genes associated with fibrosis and mineralization, thereby potentially curtailing microbial spread. In parallel, *S. mutans* instigates a similar response by triggering the MAPK/P38, MAPK/ERK1/2, NF-κB, and TGF-β/SMAD pathways. However, its distinctive effect is the activation of the JUN transcription factor, thereby driving both immune and fibrotic responses (**Figure 7**). Taken together, our research uncovers the multifaceted immune responses of DPSCs to different microbial infections, shedding new light on the intricate dynamics of oral microbe-host cell interactions and offering novel avenues for investigation.

There are a few limitations here, the first being that we carried out the co-culture of DPSCs and microbials for four hours. Various time points were required to explore the mechanism of DPSCs against microbial infection. Besides, we will isolate and culture DPSC.7 to verify gene expression and pathway activity and confirm the process of response to *C. albicans* infection through animal and cytological experiments.

## 5 Conclusions

In conclusion, leveraged single-cell RNA sequencing to delve into the cellular and molecular properties of DPSCs in response to oral fungal and bacterial infections. The identification of the DPSC.7 subcluster, which exhibits a specific reactivity to various pathogens *via* the ROS pathway, marks a significant discovery. Uncovering this connection might be the first step to opening the door to new possibilities for treating fungal infections with DPSCs.

## Data Availability

The datasets presented in this study are deposited in the National Microbiology Data Center (NMDC) repository, accession number NMDC10018945. The specific names of the repository/repositories and accession number(s) can be found in the **Supplementary Material**.
