# Supplementary material for: Specific cell subclusters of dental pulp stem cells respond to distinct pathogens through the ROS pathway
Source: Front Cell Infect Microbiol. 2024 Sep 12;14:1452124. doi: 10.3389/fcimb.2024.1452124 (PMC11424553; doi:10.3389/fcimb.2024.1452124)
Supplement: Supplementary file 1 [file DataSheet1.docx]

Supplementary Material

Specific cell subclusters of dental pulp stem cells respond to distinct pathogens through the ROS pathway

Tiansong Xu^1,2†^, Yangjia Liu^1†^, Wen Zhang^3,4†^, Murong Li^1^, Liqi Zhang^1^, Xueying Li^3^, Yifei Zhang^1^, Lin Yue^3^, Sha Li^5^, Ye Lin^5^, Xiaoying Zou^3,6*‡^, Feng Chen^1*‡^

^1^Central Laboratory, Peking University School and Hospital of Stomatology & National Center for Stomatology & National Clinical Research Center for Oral Diseases & National Engineering Research Center of Oral Biomaterials and Digital Medical Devices, Beijing, China

^2^Fifth Clinical Division, Peking University School and Hospital of Stomatology & National Center for Stomatology & National Clinical Research Center for Oral Diseases & National Engineering Research Center of Oral Biomaterials and Digital Medical Devices, Beijing, China

^3^Department of Cariology and Endodontology, Peking University School and Hospital of Stomatology & National Center for Stomatology & National Clinical Research Center for Oral Diseases & National Engineering Research Center of Oral Biomaterials and Digital Medical Devices, Beijing, China

^4^Department of Stomatology, Peking University International Hospital, Beijing, China

^5^Department of Implantology, Peking University School and Hospital of Stomatology & National Center for Stomatology & National Clinical Research Center for Oral Diseases & National Engineering Research Center of Oral Biomaterials and Digital Medical Devices, Bejing, China

^6^Center of Stomatology, Peking University Hospital, Beijing, China

† **These authors contributed equally to this work and shared the first authorship.**

‡ **These authors have contributed equally to this work and share correspondence authorship.**

# Supplementary Figures and Tables

## Supplementary Figures

**
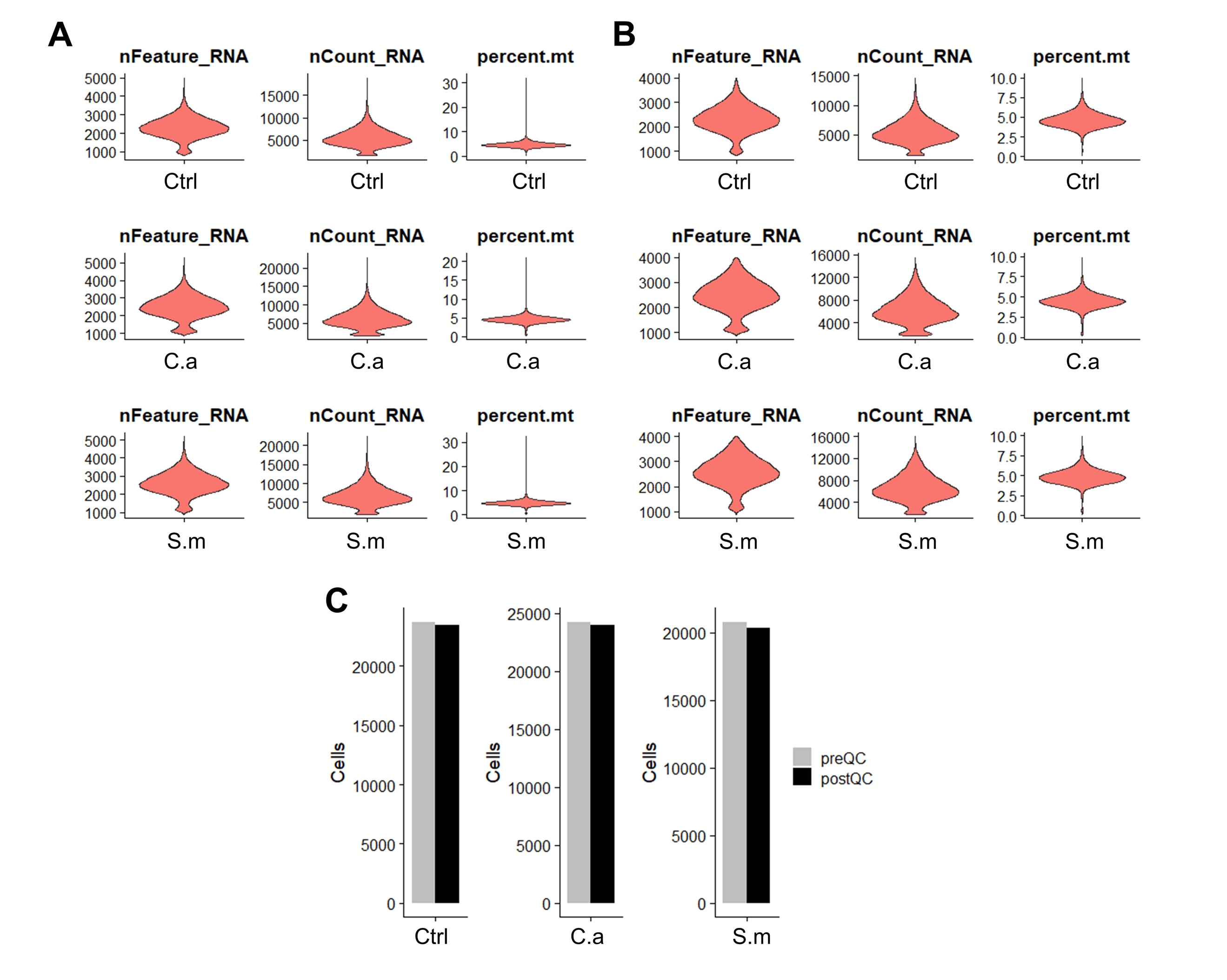
**

**Supplementary Figure S1.** Quality control related to Figure 1.

(A-B) Violin plots show feature numbers, RNA counts, and percent mitochondrial transcripts from the sample without infection (Ctrl), the sample infected by *C. albicans* (C.a), and the sample infected by *S. mutans* (S.m) before (A) and after (B) quality control. (C) Bar graphs demonstrate cell numbers before (pre-QC) and after (post-QC) quality control.

**
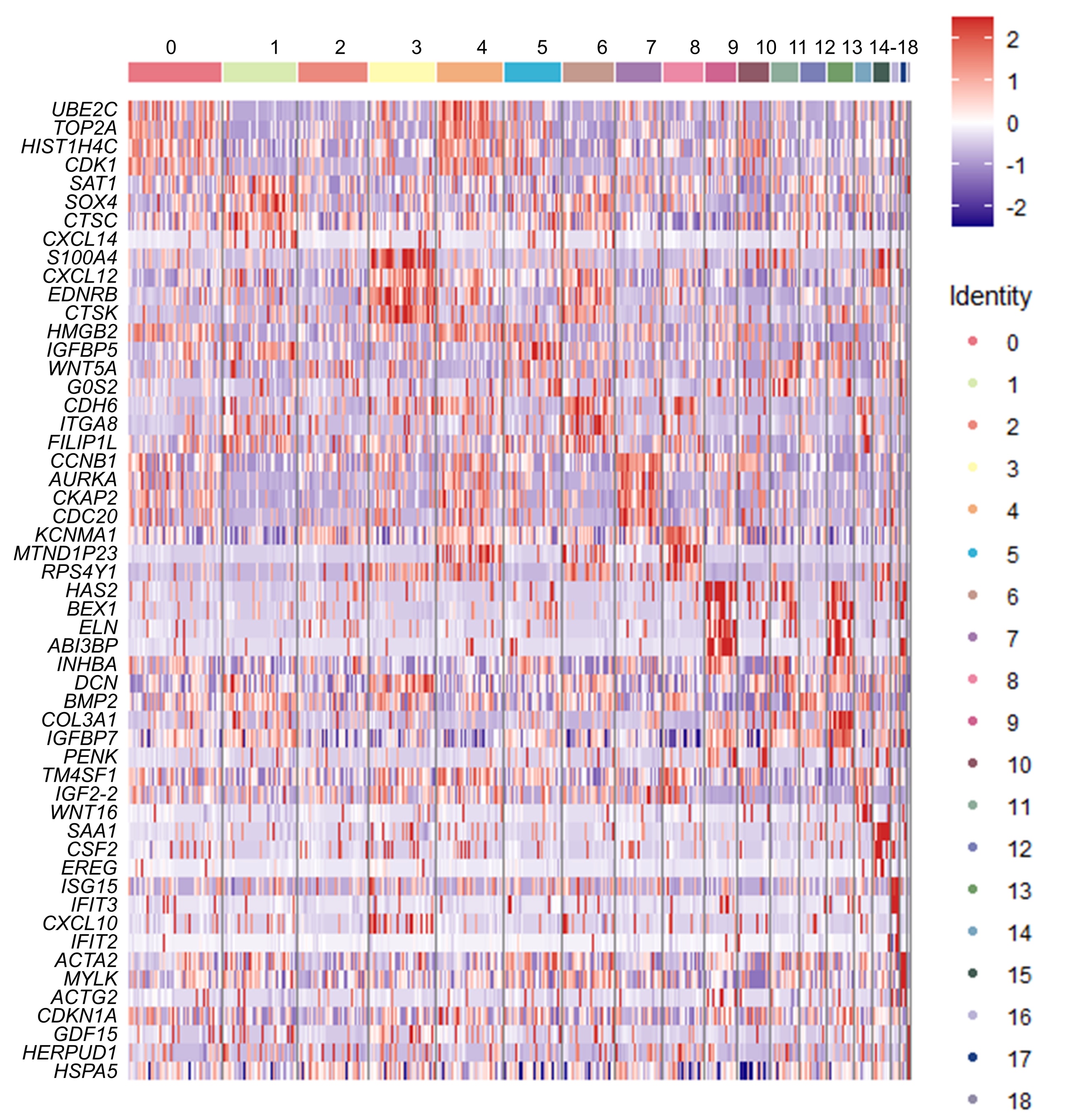
**

**Supplementary Figure S2.** The expression of marker genes in DPSCs subclusters, related to Figure 1.

Heatmap shows the differential expression of marker genes in DPSCs subclusters.


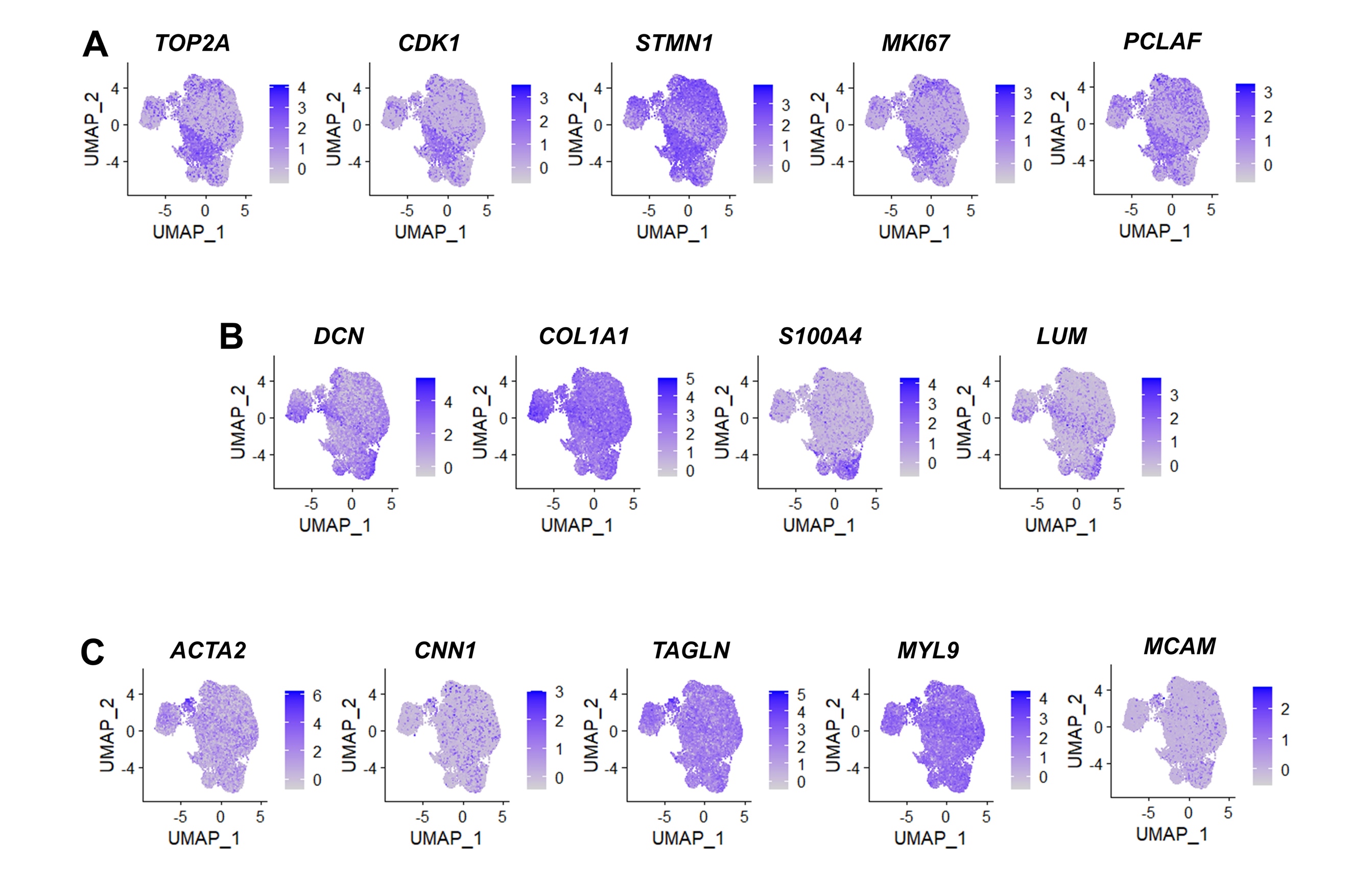


**Supplementary Figure S3.** UMAP shows the expression of genes characterizing subclusters of DPSCs, related to Figure 1.

UMAP represents the main gene markers for each subtype of classical DPSCs (*TOP2A*, *CDK1*, *STMN1*, *MKI67*, and *PCLAF*): (A), fibroblast-like DPSCs (*DCN*, *COL1A1*, *LUM*, and *S100A4*); (B), smooth muscle-like DPSCs (*ACTA2*, *TAGLN*, *MYL9*, and *CNN1*3); (C), and perivascular-like DPSCs (*ACTA2*, *MCAM*, *TAGLN*, and *MYL9*) (C).


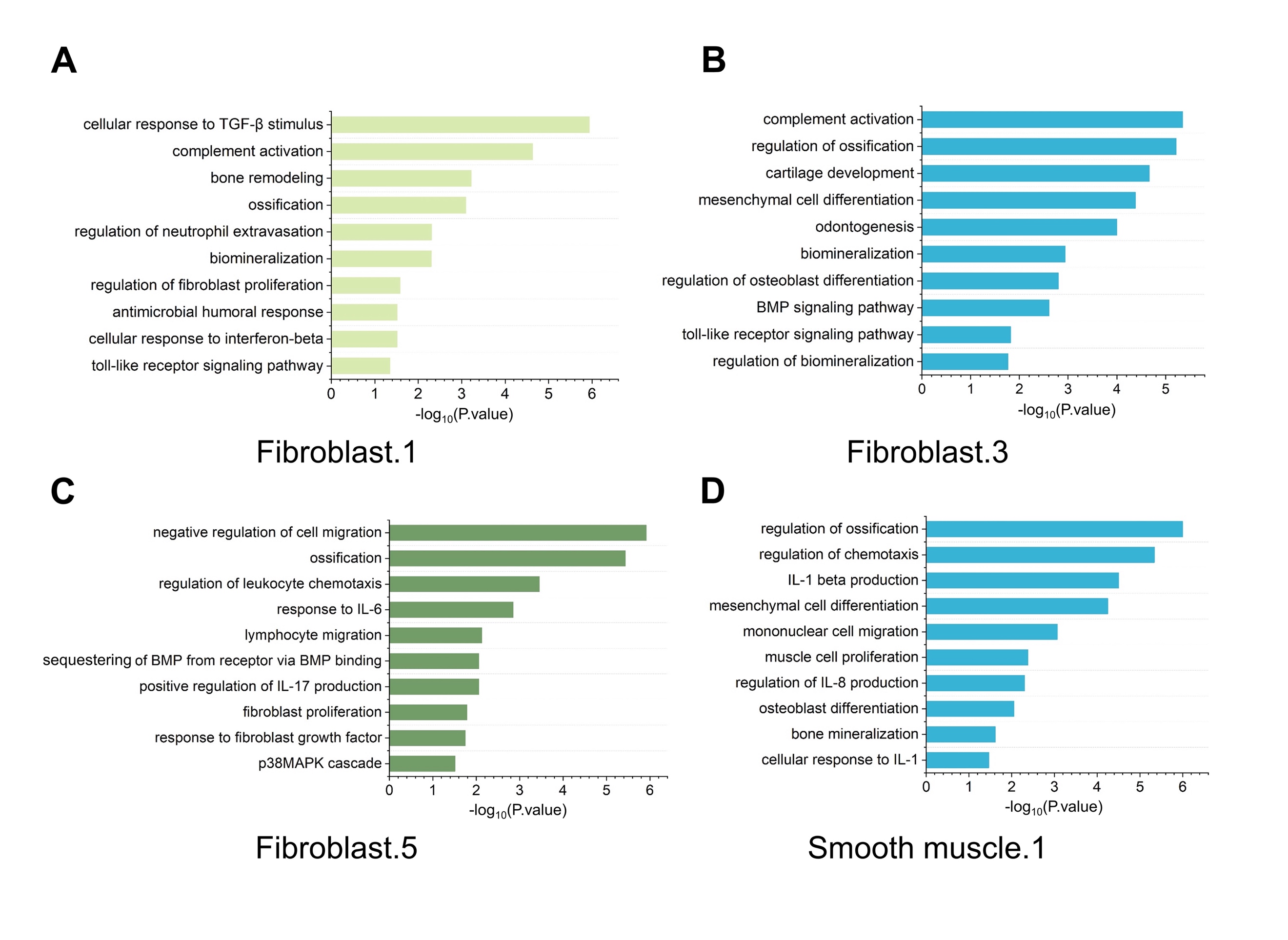


**Supplementary Figure S4.** Expression of the differential gene of DPSC.7, related to Figure 1.

Bar graphs depict GO terms enriched in Fibroblast.1 (A), Fibroblast.3 (B), Fibroblast.5 (C), and Smooth muscle.2 (D).


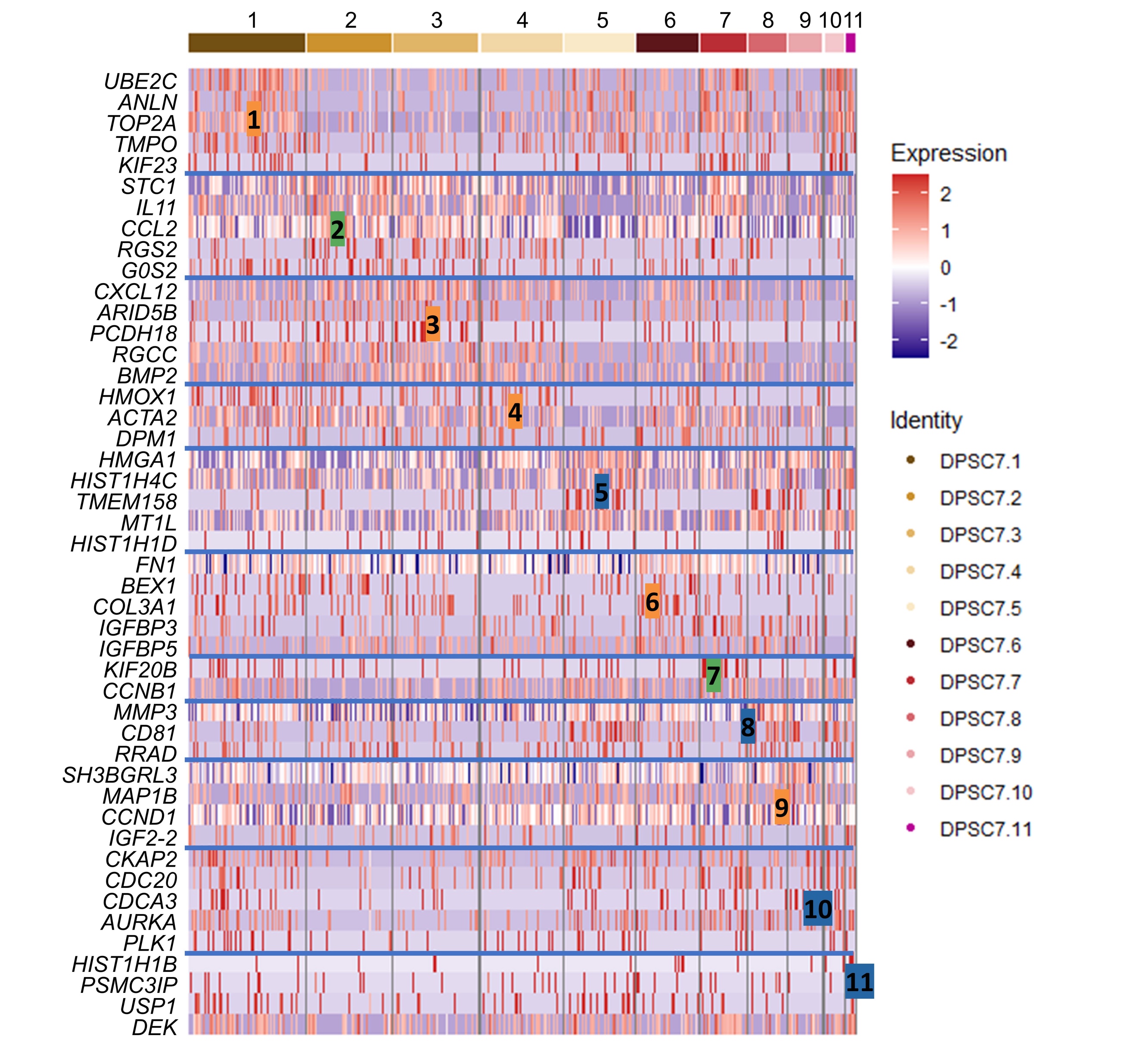


**Supplementary Figure S5.** The expression of marker genes in DPSC.7 subgroups, related to Figure 2.

Heatmap shows differential expression of marker genes of DPSCs.


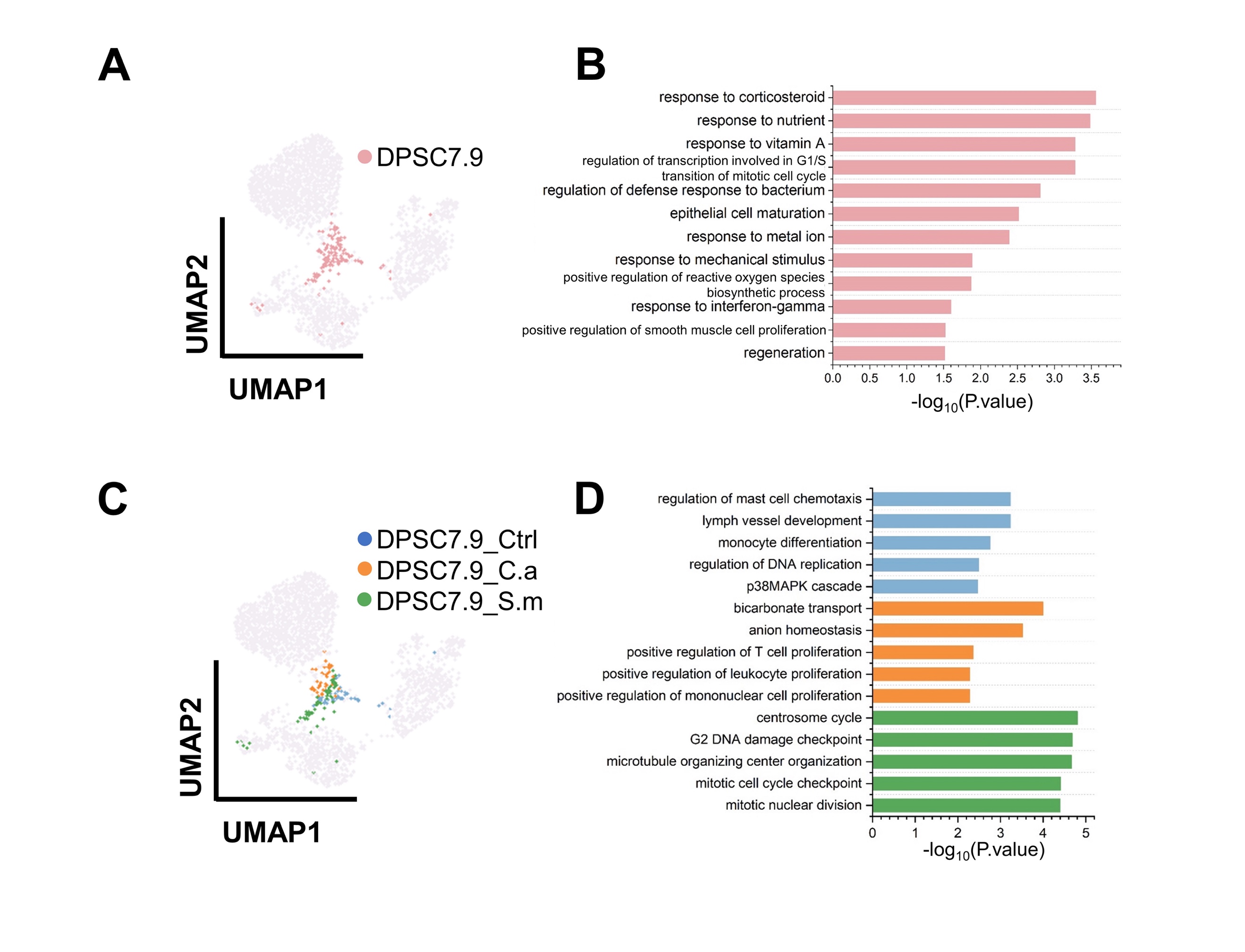


**Supplementary Figure S6.** DPSC7.9 among Ctrl, C.a, and S.m samples, related to Figure 3.

(A, C) UMAP plots depict DPSC7.9 (A) and DPSC7.9 divided into Ctrl, C.a, and S.m samples (C). (B, D) Bar plots show pathways enriched in DPSC7.9 (B) and DPSC7.9 divided into Ctrl, C.a, and S.m samples (D). The length of each bar represents the P-value from each pathway.


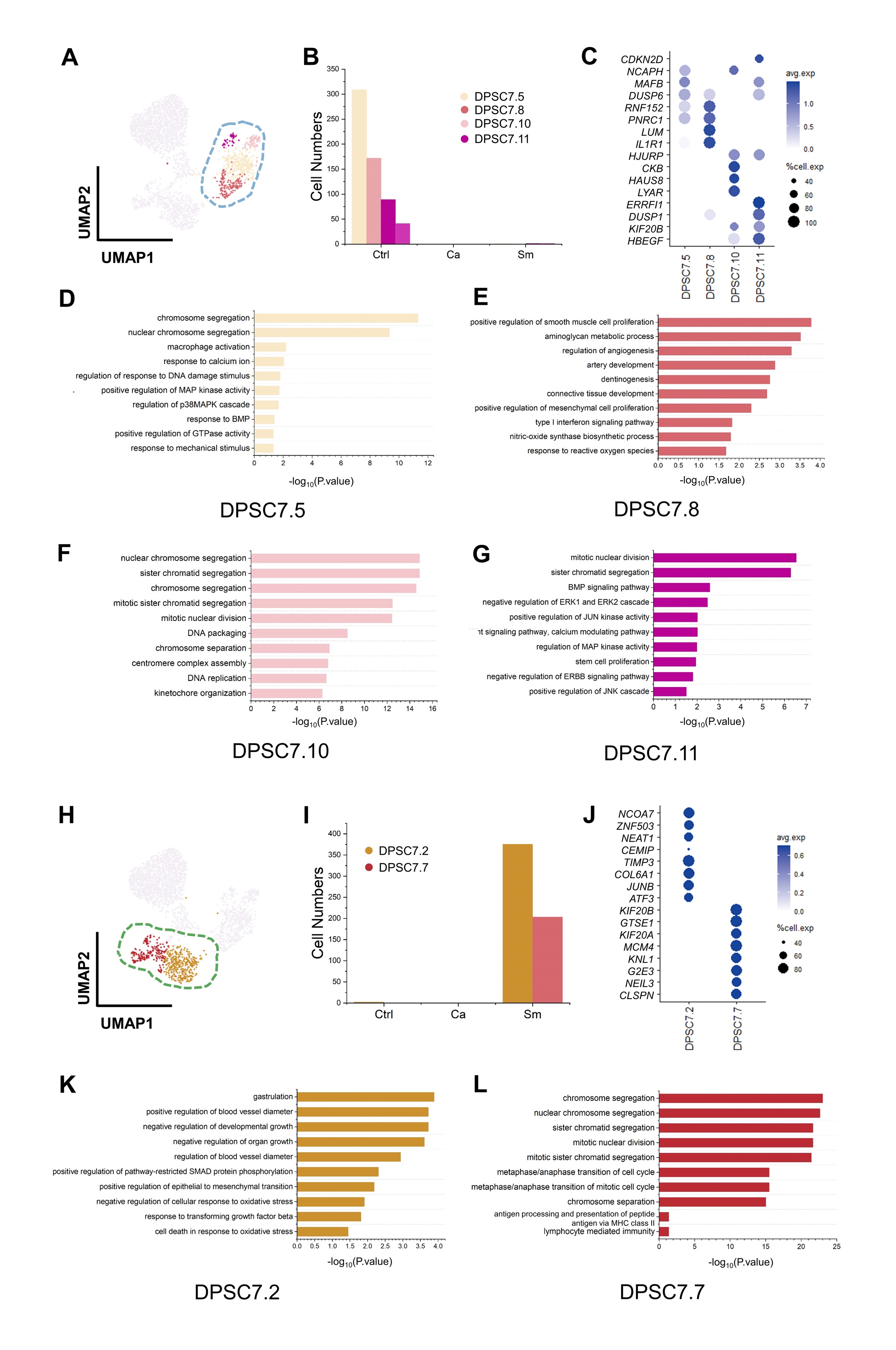


**Supplementary Figure S7.** The cell numbers and pathway enrichment of DPSC7.5, DPSC7.8, DPSC7.10, and DPSC7.11, related to Figure 3.

(A) UMAP represents DPSC7.5, DPSC7.8, DPSC7.10, and DPSC7.11. (B) Bar graphs show the cell numbers of DPSC7.5 (Ctrl [309 cells] and S.m [1 cell]), DPSC7.8 (Ctrl [172 cells]), DPSC7.10 (Ctrl [89 cells] and S.m [1 cell]), and DPSC7.11 (Ctrl [41 cells] and S.m [1 cell]). (C) Dot plots depict the expression of signature genes and the percentage of cells expressing genes involved in DPSC7.5, DPSC7.8, DPSC7.10, and DPSC7.11. (D-G) Bar plots show pathways enriched in DPSC7.5 (D), DPSC7.8 (E), DPSC7.10 (F), and DPSC7.11 (G). The length of each bar represents the P-value of pathways. (H) UMAP plot represents DPSC7.2 and DPSC7.7. (I) Bar graphs show the cell numbers of DPSC7.2 (Ctrl [2 cells] and S.m [375 cells]) and DPSC7.7 (S.m [203 cells]) in three samples. (J) Dot plots show the expression of cluster-defining genes and the percentage of chosen signature gene expressions of DPSC7.2 and DPSC7.7. (K-L) Bar graphs demonstrate the GO terms enriched in DPSC7.2 (K) and DPSC7.7 (L).


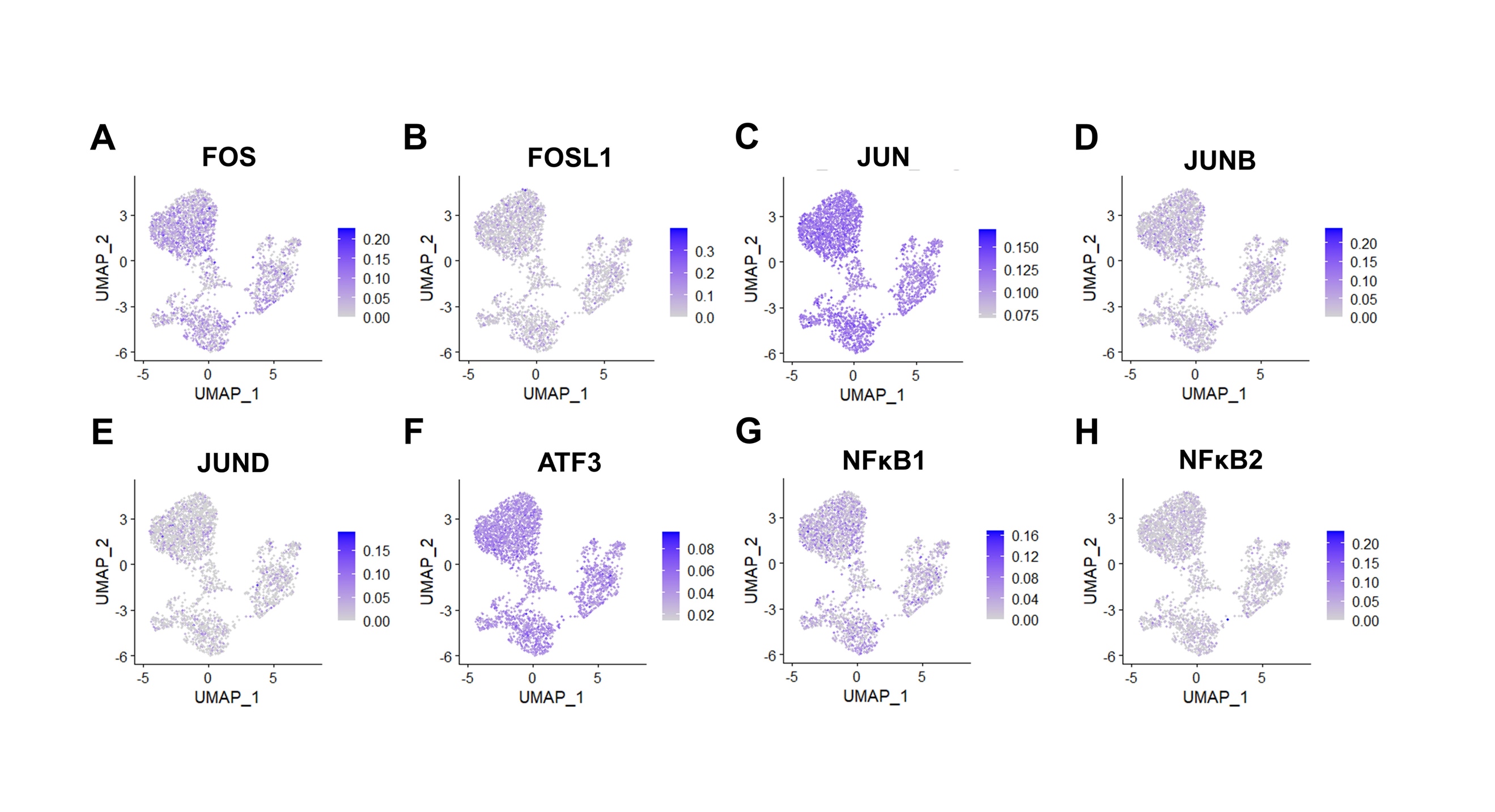


**Supplementary Figure S8.** The activity of each regulon for transcription factors in DPSC.7, related to Figure 4.

UMAP plots show AUC scores of TF motifs by SCENIC in DPSC.7, including FOS (A), FOSL1 (B), JUN (C), JUNB (D), JUND (E), ATF3 (F), NFκB1 (G), and NFκB2 (H). The data has been color-coded according to the AUC scores of each regulon above.


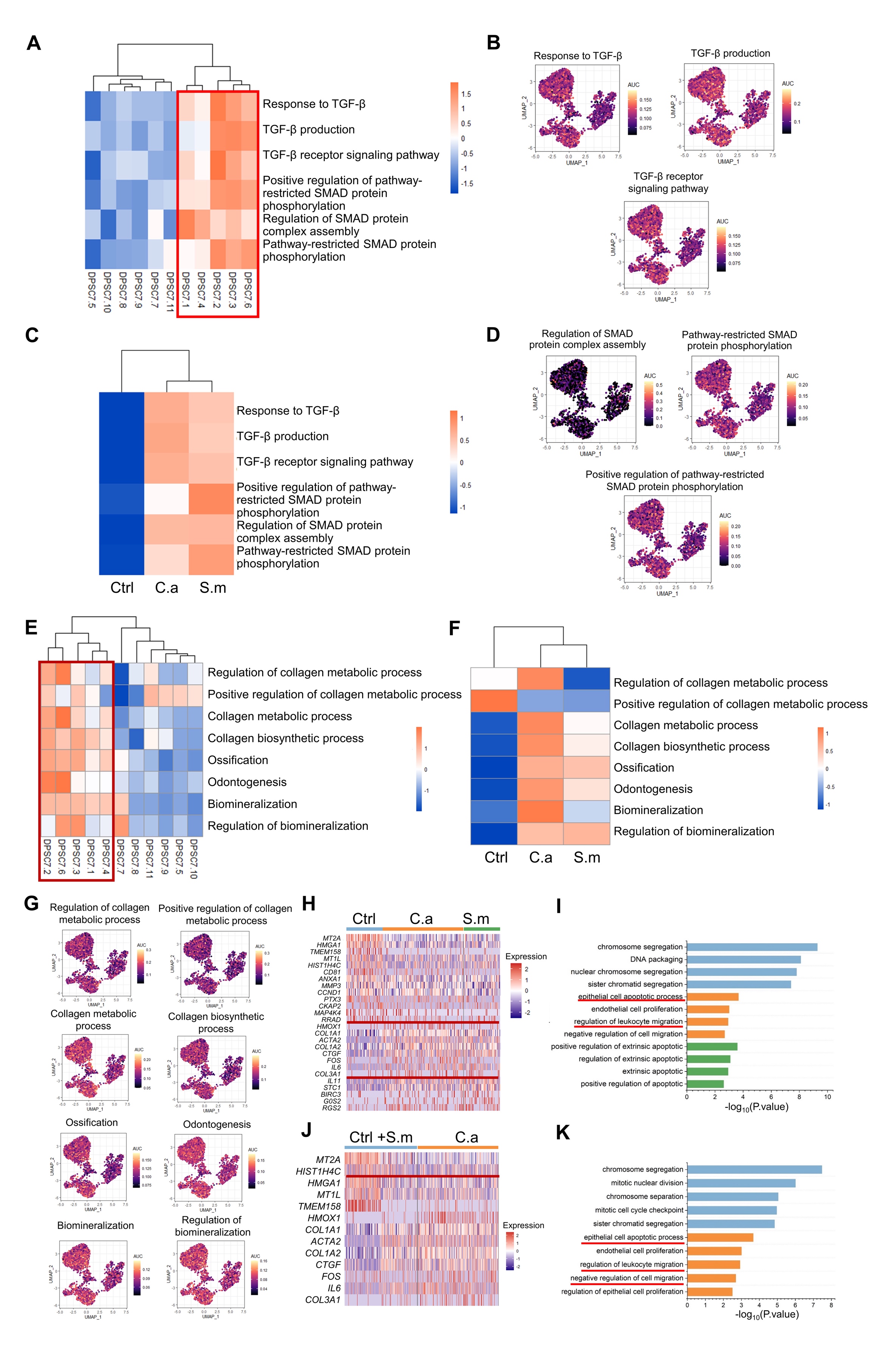


**Supplementary Figure S9.** The activity of TGF-β/SMAD pathways in the Infected sample of DPSC.7.

(A, C) Heatmaps depict GSVA scores for TGF-β/SMAD pathways among eleven subclusters (A) and three samples (C). (B, D) UMAP plots show AUC score distributions about TGF-β (B)/SMAD (D) pathways. (E-F) Heatmaps indicate the enrichment of pathways linked to collagen, odontogenesis, and biomineralization among eleven DPSC.7 clusters (E) and three samples (F). (G) UMAP shows AUC score distributions of pathways with UMAP in DPSC.7, associated with collagen, odontogenesis, and biomineralization. (H, J) Heatmaps show the average expression of the top differentially expressed genes for each cluster of DPSC.7 cells among three samples (Ctrl, C.a, and S.m) (H), and two samples (Ctrl+S.m and C.a) (J). (I, K) Bar graphs show the enriched GO terms among three samples (Ctrl, C.a and, S.m) (I) and two samples (Ctrl+S.m and C.a) (K).

## Supplementary Tables

**Supplementary Table S1.** Cell number and percentage of major cell types in each cluster after quality control and batch correction, related to Figure 1.

|  |  | **Cell number** | | | **Cell percentage** | | |
| --- | --- | --- | --- | --- | --- | --- | --- |
| **Cluster** | **Annotation** | **Ctrl** | **C.a** | **S.m** | **Ctrl** | **C.a** | **S.m** |
| 0 | DPSC.1 | 4048 | 2367 | 1893 | 17.26% | 9.86% | 9.31% |
| 1 | Fibroblast.1 | 1013 | 2958 | 2677 | 4.32% | 12.32% | 13.17% |
| 2 | DPSC.2 | 2553 | 2089 | 1606 | 10.88% | 8.70% | 7.90% |
| 3 | Fibroblast.2 | 1905 | 2157 | 1929 | 8.12% | 8.98% | 9.49% |
| 4 | DPSC.3 | 2614 | 1785 | 1587 | 11.14% | 7.44% | 7.81% |
| 5 | muscle.1 | 1664 | 1801 | 1583 | 7.09% | 7.50% | 7.79% |
| 6 | Fibroblast.3 | 577 | 2143 | 1954 | 2.46% | 8.93% | 9.61% |
| 7 | DPSC.4 | 2374 | 955 | 829 | 10.12% | 3.98% | 4.08% |
| 8 | DPSC.5 | 1436 | 1130 | 1009 | 6.12% | 4.71% | 4.96% |
| 9 | DPSC.6 | 1278 | 841 | 756 | 5.45% | 3.50% | 3.72% |
| 10 | DPSC.7 | 672 | 1507 | 680 | 2.86% | 6.28% | 3.34% |
| 11 | Fibroblast.4 | 755 | 918 | 826 | 3.22% | 3.82% | 4.06% |
| 12 | muscle.2 | 464 | 1031 | 783 | 1.98% | 4.29% | 3.85% |
| 13 | Fibroblast.5 | 489 | 920 | 828 | 2.08% | 3.83% | 4.07% |
| 14 | muscle.3 | 476 | 587 | 555 | 2.03% | 2.45% | 2.73% |
| 15 | Fibroblast.6 | 440 | 448 | 440 | 1.88% | 1.87% | 2.16% |
| 16 | DPSC.8 | 468 | 106 | 203 | 1.99% | 0.44% | 1.00% |
| 17 | Perivascular-like | 152 | 206 | 134 | 0.65% | 0.86% | 0.66% |
| 18 | DPSC.9 | 81 | 59 | 60 | 0.35% | 0.25% | 0.30% |
| Sum | | 23459 | 24008 | 20332 | 100.00% | 100.00% | 100.00% |

**Supplementary Table S2.** List of genes differentially expressed (logfc.threshold=0.25 min.pct=0.1 only.pos=T) between DPSC.7 and other clusters, related to Figure 2.

| **p_val** | **avg_log2FC** | **pct.1** | **pct.2** | **p_val_adj** | **gene** |
| --- | --- | --- | --- | --- | --- |
| 9.96E-302 | 0.409265 | 1 | 1 | 1.99E-298 | *TXN* |
| 5.07E-226 | 0.510184 | 0.997 | 1 | 1.01E-222 | *MALAT1* |
| 6.30E-48 | 0.282539 | 0.876 | 0.94 | 1.26E-44 | *MMP3* |
| 2.22E-21 | 0.437637 | 0.576 | 0.687 | 4.45E-18 | *HIST1H4C* |
| 2.94E-15 | 0.351536 | 0.835 | 0.956 | 5.87E-12 | *CCND1* |
| 4.77E-15 | 0.277745 | 0.856 | 0.971 | 9.55E-12 | *RPS27L* |

**Supplementary Table S3.** Table of gene ontology pathway related to pathways enriched in DPSC.7, related to Figure 2.

| **Geneset_id** | **Description** | **fg_freq** | **bg_freq** | **n_fg** | **n_bg** | **n_set** | **p_val** | **p_adj** |
| --- | --- | --- | --- | --- | --- | --- | --- | --- |
| GO:0071731 | response to nitric oxide | 0.4 | 0.002048 | 2 | 6 | 20 | 3.49E-05 | 0.000883 |
| GO:0034614 | cellular response to reactive oxygen species | 0.6 | 0.018089 | 3 | 53 | 184 | 5.45E-05 | 0.001213 |
| GO:0010971 | positive regulation of G2/M transition of mitotic cell cycle | 0.4 | 0.00273 | 2 | 8 | 27 | 6.50E-05 | 0.0014 |
| GO:1902751 | positive regulation of cell cycle G2/M phase transition | 0.4 | 0.003072 | 2 | 9 | 31 | 8.35E-05 | 0.001705 |
| GO:0000302 | response to reactive oxygen species | 0.6 | 0.024915 | 3 | 73 | 262 | 0.000143 | 0.002437 |
| GO:0034599 | cellular response to oxidative stress | 0.6 | 0.032082 | 3 | 94 | 374 | 0.000305 | 0.004142 |
| GO:0031571 | mitotic G1 DNA damage checkpoint | 0.4 | 0.006143 | 2 | 18 | 67 | 0.000353 | 0.004567 |
| GO:0044819 | mitotic G1/S transition checkpoint | 0.4 | 0.006143 | 2 | 18 | 70 | 0.000353 | 0.004567 |
| GO:0044783 | G1 DNA damage checkpoint | 0.4 | 0.006485 | 2 | 19 | 68 | 0.000394 | 0.004916 |
| GO:0062197 | cellular response to chemical stress | 0.6 | 0.038567 | 3 | 113 | 436 | 0.000528 | 0.006063 |

**Supplementary Table S4.** List of the signature genes of each cluster of DPSC.7 divided by unsupervised clustering, related to Figure 2.

| **p_val** | **avg_log2FC** | **pct.1** | **pct.2** | **p_val_adj** | **gene** | **cluster** |
| --- | --- | --- | --- | --- | --- | --- |
| 8.40E-19 | 0.682022 | 0.497 | 0.456 | 1.68E-15 | *ANLN* | DPSC7.1 |
| 1.33E-18 | 0.487047 | 0.788 | 0.713 | 2.66E-15 | *TUBB4B* | DPSC7.1 |
| 4.01E-18 | 0.434845 | 0.222 | 0.271 | 8.01E-15 | *CDCA3* | DPSC7.1 |
| 4.82E-17 | 0.606211 | 0.379 | 0.377 | 9.64E-14 | *TMPO* | DPSC7.1 |
| 9.85E-70 | 0.280462 | 0.891 | 0.342 | 1.97E-66 | *NCOA7* | DPSC7.2 |
| 1.90E-30 | 0.289239 | 0.775 | 0.337 | 3.79E-27 | *ZNF503* | DPSC7.2 |
| 7.97E-29 | 0.494883 | 0.719 | 0.225 | 1.59E-25 | *NEAT1* | DPSC7.2 |
| 1.03E-27 | 0.329034 | 0.379 | 0.344 | 2.06E-24 | *CEMIP* | DPSC7.2 |
| 2.02E-12 | 0.376852 | 0.96 | 0.912 | 4.03E-09 | *FGF7* | DPSC7.3 |
| 5.21E-12 | 0.341845 | 0.89 | 0.887 | 1.04E-08 | *ACSL4* | DPSC7.3 |
| 3.01E-08 | 0.377676 | 0.35 | 0.393 | 6.03E-05 | *ITGA2* | DPSC7.3 |
| 2.50E-07 | 0.267138 | 0.179 | 0.478 | 0.00050034 | *ITGA8* | DPSC7.3 |
| 1.63E-18 | 0.254601 | 0.102 | 0.479 | 3.25E-15 | *CXCL14* | DPSC7.4 |
| 2.38E-14 | 0.250415 | 0.184 | 0.532 | 4.76E-11 | *CSF2* | DPSC7.4 |
| 3.48E-06 | 0.323765 | 0.637 | 0.641 | 0.00695408 | *RANBP1* | DPSC7.4 |
| 3.95E-06 | 0.416683 | 0.357 | 0.657 | 0.00789891 | *HMOX1* | DPSC7.4 |
| 7.59E-115 | 0.384387 | 0.961 | 0.293 | 1.52E-111 | *CDKN2D* | DPSC7.5 |
| 1.11E-81 | 0.548556 | 0.89 | 0.285 | 2.23E-78 | *NCAPH* | DPSC7.5 |
| 4.77E-80 | 0.264248 | 0.868 | 0.282 | 9.54E-77 | *MAFB* | DPSC7.5 |
| 2.30E-77 | 0.262279 | 0.997 | 0.44 | 4.61E-74 | *DUSP6* | DPSC7.5 |
| 1.20E-19 | 0.605644 | 0.978 | 0.944 | 2.41E-16 | *FN1* | DPSC7.6 |
| 3.01E-15 | 0.262375 | 1 | 1 | 6.02E-12 | *FTL* | DPSC7.6 |
| 9.67E-15 | 0.446421 | 0.963 | 0.933 | 1.93E-11 | *SPARC* | DPSC7.6 |
| 1.45E-13 | 0.329165 | 0.996 | 0.993 | 2.90E-10 | *TGFBI* | DPSC7.6 |
| 9.50E-62 | 0.931803 | 0.936 | 0.313 | 1.90E-58 | *KIF20B* | DPSC7.7 |
| 6.11E-57 | 0.696586 | 0.956 | 0.393 | 1.22E-53 | *GTSE1* | DPSC7.7 |
| 2.50E-46 | 0.45818 | 0.818 | 0.223 | 5.00E-43 | *KIF20A* | DPSC7.7 |
| 3.67E-46 | 0.587978 | 0.926 | 0.316 | 7.33E-43 | *MCM4* | DPSC7.7 |
| 1.20E-56 | 0.303855 | 1 | 0.336 | 2.40E-53 | *RNF152* | DPSC7.8 |
| 2.54E-50 | 0.379512 | 0.983 | 0.429 | 5.08E-47 | *PNRC1* | DPSC7.8 |
| 3.22E-49 | 0.33871 | 1 | 0.439 | 6.44E-46 | *LUM* | DPSC7.8 |
| 8.21E-39 | 0.38303 | 0.936 | 0.364 | 1.64E-35 | *EXOSC9* | DPSC7.8 |
| 4.93E-44 | 0.294528 | 0.736 | 0.285 | 9.87E-41 | *ITGA11* | DPSC7.9 |
| 1.95E-37 | 0.471064 | 0.736 | 0.286 | 3.91E-34 | *HIST1H2BC* | DPSC7.9 |
| 7.37E-35 | 0.359989 | 0.75 | 0.36 | 1.47E-31 | *MTND1P23* | DPSC7.9 |
| 1.19E-32 | 0.262074 | 0.764 | 0.388 | 2.39E-29 | *IGFBP2* | DPSC7.9 |
| 2.14E-23 | 0.756019 | 0.889 | 0.368 | 4.28E-20 | *HJURP* | DPSC7.10 |
| 5.45E-23 | 0.302719 | 0.944 | 0.382 | 1.09E-19 | *CKB* | DPSC7.10 |
| 6.50E-21 | 0.319565 | 0.789 | 0.233 | 1.30E-17 | *HAUS8* | DPSC7.10 |
| 1.94E-20 | 0.425417 | 0.956 | 0.395 | 3.88E-17 | *LYAR* | DPSC7.10 |
| 3.49E-17 | 0.704329 | 1 | 0.47 | 6.98E-14 | *ERRFI1* | DPSC7.11 |
| 6.27E-15 | 0.329412 | 1 | 0.424 | 1.25E-11 | *DUSP1* | DPSC7.11 |
| 2.28E-12 | 0.479419 | 0.929 | 0.349 | 4.57E-09 | *KIF20B* | DPSC7.11 |
| 2.59E-12 | 0.41217 | 0.976 | 0.471 | 5.18E-09 | *HBEGF* | DPSC7.11 |

**Supplementary Table S5.** Table of Gene Ontology pathways related to pathways enriched in DPSC7.1, DPSC7.3, DPSC7.4, and DPSC7.6, which predominantly consisted of cells in C.a sample, related to Figure 3.

| **Geneset_id** | **Description** | **fg_freq** | **bg_freq** | **n_fg** | **n_bg** | **n_set** | **p_val** | **cluster** | **p_adj** |
| --- | --- | --- | --- | --- | --- | --- | --- | --- | --- |
| GO:0140014 | mitotic nuclear division | 0.55 | 0.029935 | 11 | 37 | 394 | 4.88E-13 | DPSC7.1 | 6.96E-11 |
| GO:0007088 | regulation of mitotic nuclear division | 0.4 | 0.017799 | 8 | 22 | 195 | 2.70E-10 | DPSC7.1 | 2.36E-08 |
| GO:0051783 | regulation of nuclear division | 0.4 | 0.020227 | 8 | 25 | 227 | 8.89E-10 | DPSC7.1 | 7.01E-08 |
| GO:0000819 | sister chromatid segregation | 0.35 | 0.01699 | 7 | 21 | 281 | 9.20E-09 | DPSC7.1 | 6.15E-07 |
| GO:0007059 | chromosome segregation | 0.4 | 0.026699 | 8 | 33 | 488 | 1.06E-08 | DPSC7.1 | 6.87E-07 |
| GO:0098813 | nuclear chromosome segregation | 0.35 | 0.019417 | 7 | 24 | 400 | 2.66E-08 | DPSC7.1 | 1.64E-06 |
| GO:0000070 | mitotic sister chromatid segregation | 0.3 | 0.014563 | 6 | 18 | 228 | 1.31E-07 | DPSC7.1 | 7.16E-06 |
| GO:0000077 | DNA damage checkpoint | 0.1 | 0.012136 | 2 | 15 | 179 | 0.023032 | DPSC7.1 | 0.06137 |
| GO:0021700 | developmental maturation | 0.1 | 0.01699 | 2 | 21 | 298 | 0.043467 | DPSC7.1 | 0.092939 |
| GO:0009612 | response to mechanical stimulus | 0.1 | 0.017799 | 2 | 22 | 226 | 0.047354 | DPSC7.1 | 0.099982 |
| GO:0044706 | multi-multicellular organism process | 0.5 | 0.025183 | 3 | 31 | 238 | 0.000275 | DPSC7.3 | 0.003425 |
| GO:0015908 | fatty acid transport | 0.333333 | 0.004874 | 2 | 6 | 116 | 0.000295 | DPSC7.3 | 0.003499 |
| GO:0048333 | mesodermal cell differentiation | 0.333333 | 0.004874 | 2 | 6 | 37 | 0.000295 | DPSC7.3 | 0.003499 |
| GO:0015718 | monocarboxylic acid transport | 0.333333 | 0.006499 | 2 | 8 | 224 | 0.000548 | DPSC7.3 | 0.005678 |
| GO:0001707 | mesoderm formation | 0.333333 | 0.007311 | 2 | 9 | 81 | 0.000703 | DPSC7.3 | 0.006831 |
| GO:0007179 | transforming growth factor beta receptor signaling pathway | 0.333333 | 0.022746 | 2 | 28 | 288 | 0.007077 | DPSC7.3 | 0.027938 |
| GO:0071496 | cellular response to external stimulus | 0.333333 | 0.02437 | 2 | 30 | 374 | 0.008108 | DPSC7.3 | 0.031412 |
| GO:0010632 | regulation of epithelial cell migration | 0.333333 | 0.026807 | 2 | 33 | 365 | 0.009777 | DPSC7.3 | 0.034739 |
| GO:0071559 | response to transforming growth factor beta | 0.333333 | 0.026807 | 2 | 33 | 353 | 0.009777 | DPSC7.3 | 0.034739 |
| GO:0010631 | epithelial cell migration | 0.333333 | 0.034119 | 2 | 42 | 452 | 0.015631 | DPSC7.3 | 0.047505 |
| GO:2001237 | negative regulation of extrinsic apoptotic signaling pathway | 0.5 | 0.011373 | 2 | 14 | 123 | 0.000712 | DPSC7.4 | 0.006831 |
| GO:2001236 | regulation of extrinsic apoptotic signaling pathway | 0.5 | 0.017872 | 2 | 22 | 194 | 0.001791 | DPSC7.4 | 0.011879 |
| GO:0002685 | regulation of leukocyte migration | 0.5 | 0.021121 | 2 | 26 | 252 | 0.002509 | DPSC7.4 | 0.015132 |
| GO:0097191 | extrinsic apoptotic signaling pathway | 0.5 | 0.021121 | 2 | 26 | 306 | 0.002509 | DPSC7.4 | 0.015132 |
| GO:2001234 | negative regulation of apoptotic signaling pathway | 0.5 | 0.032494 | 2 | 40 | 288 | 0.00593 | DPSC7.4 | 0.024566 |
| GO:0030336 | negative regulation of cell migration | 0.411765 | 0.026829 | 7 | 33 | 399 | 8.29E-08 | DPSC7.6 | 4.74E-06 |
| GO:0007162 | negative regulation of cell adhesion | 0.294118 | 0.025203 | 5 | 31 | 348 | 3.65E-05 | DPSC7.6 | 0.000773 |
| GO:0010812 | negative regulation of cell-substrate adhesion | 0.176471 | 0.00813 | 3 | 10 | 70 | 0.000248 | DPSC7.6 | 0.00324 |
| GO:0071774 | response to fibroblast growth factor | 0.235294 | 0.021138 | 4 | 26 | 226 | 0.000311 | DPSC7.6 | 0.003582 |
| GO:0001503 | ossification | 0.294118 | 0.045528 | 5 | 56 | 487 | 0.000665 | DPSC7.6 | 0.006635 |
| GO:0042476 | odontogenesis | 0.176471 | 0.013008 | 3 | 16 | 150 | 0.001101 | DPSC7.6 | 0.009328 |
| GO:0007259 | receptor signaling pathway via JAK-STAT | 0.117647 | 0.008943 | 2 | 11 | 208 | 0.009195 | DPSC7.6 | 0.034399 |
| GO:0071559 | response to transforming growth factor beta | 0.176471 | 0.026829 | 3 | 33 | 353 | 0.009264 | DPSC7.6 | 0.034399 |
| GO:0031214 | biomineral tissue development | 0.117647 | 0.013821 | 2 | 17 | 203 | 0.021654 | DPSC7.6 | 0.059227 |
| GO:0032963 | collagen metabolic process | 0.117647 | 0.013821 | 2 | 17 | 140 | 0.021654 | DPSC7.6 | 0.059227 |
| GO:0070372 | regulation of ERK1 and ERK2 cascade | 0.117647 | 0.017073 | 2 | 21 | 358 | 0.032369 | DPSC7.6 | 0.076721 |

**Supplementary Table S6.** Expression of the genes associated with AP-1 transcription factors of each cluster of DPSC.7, including FOS, FOSL1, JUN, JUNB, related to Figure 4.

| **p_val** | **avg_log_2_FC** | **pct.1** | **pct.2** | **p_val_adj** | **cluster** | **gene** |
| --- | --- | --- | --- | --- | --- | --- |
| 1.01E-05 | 0.31423 | 0.308 | 0.414 | 0.020218863 | DPSC7.1 | *FOSL1* |
| 7.87E-24 | 0.194888 | 0.844 | 0.421 | 1.57E-20 | DPSC7.2 | *JUNB* |
| 2.46E-05 | 0.276914 | 0.39 | 0.671 | 0.049105196 | DPSC7.3 | *FOS* |
| 4.78E-08 | 0.077606 | 0.206 | 0.517 | 9.55E-05 | DPSC7.4 | *JUNB* |
| 0.001131128 | 0.187869 | 0.297 | 0.409 | 1 | DPSC7.4 | *FOSL1* |
| 9.92E-40 | 0.536395 | 0.99 | 0.557 | 1.98E-36 | DPSC7.5 | *JUN* |
| 0.009966273 | 0.249933 | 0.426 | 0.656 | 1 | DPSC7.6 | *FOS* |
| 3.36E-19 | 0.278537 | 0.878 | 0.451 | 6.72E-16 | DPSC7.8 | *JUNB* |
| 8.64E-19 | 0.222385 | 1 | 0.611 | 1.73E-15 | DPSC7.8 | *FOS* |
| 3.60E-16 | 0.245778 | 0.988 | 0.579 | 7.20E-13 | DPSC7.8 | *JUN* |
| 5.67E-06 | 0.572703 | 0.733 | 0.384 | 0.011342707 | DPSC7.10 | *FOSL1* |
| 0.000378307 | 0.06702 | 0.857 | 0.471 | 0.756614101 | DPSC7.11 | *JUNB* |
| 0.006227987 | 0.074012 | 0.976 | 0.629 | 1 | DPSC7.11 | *FOS* |

**Supplementary Table S7.** Table of the annotated GO terms of each cluster in DPSC.7, related to Figure 5D.

| **Geneset_id** | **Description** | **GeneRatio** | **BgRatio** | **p_val** | **p_adj** | **qvalue** | **cluster** |
| --- | --- | --- | --- | --- | --- | --- | --- |
| GO:0031498 | chromatin disassembly | 1/3 | 20/18866 | 0.003177 | 0.01871 | 0.002973 | 1 |
| GO:0035455 | response to interferon-alpha | 1/11 | 21/18866 | 0.01218 | 0.093572 | 0.058943 | 2 |
| GO:1903409 | ROS biosynthetic process | 1/11 | 128/18866 | 0.072169 | 0.134539 | 0.084749 | 2 |
| GO:1990868 | response to chemokine | 5/28 | 97/18866 | 2.90E-07 | 6.32E-05 | 3.58E-05 | 3 |
| GO:0001666 | response to hypoxia | 7/28 | 359/18866 | 7.15E-07 | 9.38E-05 | 5.32E-05 | 3 |
| GO:0030199 | collagen fibril organization | 4/28 | 55/18866 | 1.26E-06 | 0.000125 | 7.08E-05 | 3 |
| GO:0071559 | response to TGF-β | 4/28 | 258/18866 | 0.00054 | 0.007169 | 0.004065 | 3 |
| GO:0070372 | regulation of ERK1 and ERK2 cascade | 4/28 | 306/18866 | 0.001022 | 0.011015 | 0.006245 | 3 |
| GO:0006979 | response to oxidative stress | 3/28 | 458/18866 | 0.029706 | 0.087852 | 0.049813 | 3 |
| GO:0034505 | tooth mineralization | 1/28 | 24/18866 | 0.035039 | 0.092508 | 0.052454 | 3 |
| GO:0038066 | p38MAPK cascade | 1/28 | 52/18866 | 0.074423 | 0.13024 | 0.073848 | 3 |
| GO:0034614 | cellular response to ROS | 1/28 | 170/18866 | 0.224022 | 0.257812 | 0.146183 | 3 |
| GO:0140014 | mitotic nuclear division | 21/53 | 286/18866 | 6.12E-25 | 7.08E-22 | 4.81E-22 | 4 |
| GO:0000280 | nuclear division | 23/53 | 428/18866 | 2.82E-24 | 1.63E-21 | 1.11E-21 | 4 |

**Supplementary Table S8.** List of the annotated GO terms of DPSC7.1, DPSC7.3, DPSC7.4, and DPSC7.6, related to Figure 5I.

| **ID** | **Description** | **GeneRatio** | **BgRatio** | **p_val** | **p_adj** | **qvalue** | **cluster** |
| --- | --- | --- | --- | --- | --- | --- | --- |
| GO:0140014 | mitotic nuclear division | 31/60 | 286/18866 | 5.91E-41 | 6.46E-38 | 4.25E-38 | 1 |
| GO:0000280 | nuclear division | 34/60 | 428/18866 | 1.37E-40 | 7.49E-38 | 4.92E-38 | 1 |
| GO:0000302 | response to ROS | 4/60 | 235/18866 | 0.006633 | 0.03194 | 0.021009 | 1 |
| GO:0034614 | cellular response to ROS | 3/60 | 170/18866 | 0.016903 | 0.065982 | 0.043401 | 1 |
| GO:0001503 | ossification | 4/20 | 412/18866 | 0.000823 | 0.017749 | 0.011472 | 2 |
| GO:0030199 | collagen fibril organization | 2/20 | 55/18866 | 0.001533 | 0.027803 | 0.017971 | 2 |
| GO:0042476 | odontogenesis | 2/20 | 132/18866 | 0.0085 | 0.077082 | 0.049822 | 2 |
| GO:0110148 | biomineralization | 2/20 | 163/18866 | 0.012728 | 0.086814 | 0.056112 | 2 |
| GO:0070371 | ERK1 and ERK2 cascade | 2/20 | 325/18866 | 0.045834 | 0.124831 | 0.080685 | 2 |
| GO:0033151 | V(D)J recombination | 2/17 | 15/18866 | 7.97E-05 | 0.023613 | 0.016571 | 3 |
| GO:0006309 | apoptotic DNA fragmentation | 2/17 | 26/18866 | 0.000245 | 0.033696 | 0.023646 | 3 |
| GO:0032872 | regulation of stress-activated MAPK cascade | 2/17 | 237/18866 | 0.018877 | 0.126395 | 0.088698 | 3 |
| GO:0051403 | stress-activated MAPK cascade | 2/17 | 286/18866 | 0.026808 | 0.134434 | 0.09434 | 3 |
| GO:0045088 | regulation of innate immune response | 2/17 | 307/18866 | 0.030558 | 0.139249 | 0.097719 | 3 |

**Supplementary Table S9.** Table showing the number of cells expressing *IL6*, *CCL2*, and *CXCL2*, related to Figure 6.

| **Cell numbers** | | **Ctrl** | **C.a** | **S.m** |
| --- | --- | --- | --- | --- |
| *IL6* | (-) | 510 | 720 | 343 |
|  | (+) | 162 | 787 | 337 |
|  | (+/-) | 1.06 | 7.02 | 5.73 |
| *CCL2* | (-) | 327 | 188 | 101 |
|  | (+) | 345 | 1319 | 579 |
|  | (+/-) | 0.32 | 1.09 | 0.98 |
| *CXCL2* | (-) | 428 | 667 | 327 |
|  | (+) | 244 | 840 | 353 |
|  | (+/-) | 0.57 | 1.26 | 1.08 |

**Supplementary Table S10.** Table of GO biological process terms related to pathways enriched in DPSC7.9, which were distributed in Ctrl, C.a, and S.m samples, related to Supplementary Figure S6B.

| **Geneset_id** | **Description** | **fg_freq** | **bg_freq** | **n_fg** | **n_bg** | **n_set** | **p_val** | **p_adj** |
| --- | --- | --- | --- | --- | --- | --- | --- | --- |
| GO:0031960 | response to corticosteroid | 0.172414 | 0.021757 | 5 | 27 | 167 | 0.000275 | 0.003425 |
| GO:0007584 | response to nutrient | 0.172414 | 0.022562 | 5 | 28 | 183 | 0.000329 | 0.00369 |
| GO:0000083 | regulation of transcription involved in G1/S transition of mitotic cell cycle | 0.068966 | 0.001612 | 2 | 2 | 37 | 0.000528 | 0.005565 |
| GO:0033189 | response to vitamin A | 0.068966 | 0.001612 | 2 | 2 | 19 | 0.000528 | 0.005565 |
| GO:1900424 | regulation of defense response to bacterium | 0.068966 | 0.002417 | 2 | 3 | 21 | 0.00156 | 0.011248 |
| GO:0002070 | epithelial cell maturation | 0.068966 | 0.003223 | 2 | 4 | 16 | 0.003075 | 0.016913 |
| GO:0010038 | response to metal ion | 0.172414 | 0.038678 | 5 | 48 | 418 | 0.004132 | 0.020656 |
| GO:0009612 | response to mechanical stimulus | 0.103448 | 0.017728 | 3 | 22 | 226 | 0.013113 | 0.042289 |
| GO:1903428 | positive regulation of ROS biosynthetic process | 0.068966 | 0.006446 | 2 | 8 | 64 | 0.013537 | 0.043511 |
| GO:0034341 | response to interferon-gamma | 0.103448 | 0.022562 | 3 | 28 | 234 | 0.025387 | 0.065484 |
| GO:0048661 | positive regulation of smooth muscle cell proliferation | 0.068966 | 0.00967 | 2 | 12 | 114 | 0.03011 | 0.074129 |
| GO:0031099 | regeneration | 0.103448 | 0.024174 | 3 | 30 | 204 | 0.030492 | 0.074974 |

**Supplementary Table S11.** Table of GO biological process terms linked with pathways enriched in DPSC7.9-Ctrl, DPSC7.9-C.a, and DPSC7.9-S.m, related to Supplementary Figure S6D.

| **Geneset_id** | **Description** | **fg_freq** | **bg_freq** | **n_fg** | **n_bg** | **n_set** | **p_val** | **cluster** | **p_adj** |
| --- | --- | --- | --- | --- | --- | --- | --- | --- | --- |
| GO:0001945 | lymph vessel development | 0.064516 | 0.001586 | 2 | 2 | 36 | 0.000585 | DPSC7.9-Ctrl | 0.010027 |
| GO:0060753 | regulation of mast cell chemotaxis | 0.064516 | 0.001586 | 2 | 2 | 9 | 0.000585 | DPSC7.9-Ctrl | 0.010027 |
| GO:0030224 | monocyte differentiation | 0.064516 | 0.002379 | 2 | 3 | 40 | 0.001729 | DPSC7.9-Ctrl | 0.012386 |
| GO:0006275 | regulation of DNA replication | 0.096774 | 0.010309 | 3 | 13 | 131 | 0.00326 | DPSC7.9-Ctrl | 0.015779 |
| GO:0038066 | p38MAPK cascade | 0.064516 | 0.003172 | 2 | 4 | 59 | 0.003405 | DPSC7.9-Ctrl | 0.015779 |
| GO:0015701 | bicarbonate transport | 0.153846 | 0.001608 | 2 | 2 | 62 | 0.000101 | DPSC7.9-C.a | 0.003975 |
| GO:0055081 | anion homeostasis | 0.153846 | 0.002412 | 2 | 3 | 71 | 0.000301 | DPSC7.9-C.a | 0.007409 |
| GO:0042102 | positive regulation of T cell proliferation | 0.153846 | 0.008039 | 2 | 10 | 114 | 0.00433 | DPSC7.9-C.a | 0.018543 |
| GO:0032946 | positive regulation of mononuclear cell proliferation | 0.153846 | 0.008842 | 2 | 11 | 162 | 0.005261 | DPSC7.9-C.a | 0.019555 |
| GO:0070665 | positive regulation of leukocyte proliferation | 0.153846 | 0.008842 | 2 | 11 | 175 | 0.005261 | DPSC7.9-C.a | 0.019555 |
| GO:0007098 | centrosome cycle | 0.235294 | 0.010417 | 4 | 13 | 174 | 1.57E-05 | DPSC7.9-S.m | 0.00286 |
| GO:0031572 | G2 DNA damage checkpoint | 0.176471 | 0.004006 | 3 | 5 | 40 | 2.07E-05 | DPSC7.9-S.m | 0.00286 |
| GO:0031023 | microtubule organizing center organization | 0.235294 | 0.011218 | 4 | 14 | 192 | 2.18E-05 | DPSC7.9-S.m | 0.00286 |
| GO:0007093 | mitotic cell cycle checkpoint | 0.235294 | 0.012821 | 4 | 16 | 210 | 3.89E-05 | DPSC7.9-S.m | 0.003151 |
| GO:0140014 | mitotic nuclear division | 0.294118 | 0.025641 | 5 | 32 | 394 | 4.00E-05 | DPSC7.9-S.m | 0.003151 |

**Supplementary Table S12.** Table of gene ontology pathway related to pathways enriched in DPSC7.2 and DPSC7.7, which were mainly from cells in the S.m sample, related to Supplementary Figure S7K-S7L.

| **Geneset_id** | **Description** | **fg_freq** | **bg_freq** | **n_fg** | **n_bg** | **n_set** | **p_val** | **cluster** | **p_adj** |
| --- | --- | --- | --- | --- | --- | --- | --- | --- | --- |
| GO:0007369 | gastrulation | 0.2 | 0.014504 | 4 | 18 | 212 | 0.00013 | DPSC7.2 | 0.002097 |
| GO:0048640 | negative regulation of developmental growth | 0.15 | 0.006446 | 3 | 8 | 118 | 0.000191 | DPSC7.2 | 0.002779 |
| GO:0097755 | positive regulation of blood vessel diameter | 0.15 | 0.006446 | 3 | 8 | 63 | 0.000191 | DPSC7.2 | 0.002779 |
| GO:0046621 | negative regulation of organ growth | 0.1 | 0.001612 | 2 | 2 | 43 | 0.000247 | DPSC7.2 | 0.00324 |
| GO:0097746 | regulation of blood vessel diameter | 0.15 | 0.011281 | 3 | 14 | 167 | 0.001165 | DPSC7.2 | 0.009656 |
| GO:0010862 | positive regulation of pathway-restricted SMAD protein phosphorylation | 0.1 | 0.005641 | 2 | 7 | 68 | 0.00494 | DPSC7.2 | 0.021665 |
| GO:0010718 | positive regulation of epithelial to mesenchymal transition | 0.1 | 0.006446 | 2 | 8 | 61 | 0.006523 | DPSC7.2 | 0.026412 |
| GO:1900408 | negative regulation of cellular response to oxidative stress | 0.1 | 0.008864 | 2 | 11 | 67 | 0.012445 | DPSC7.2 | 0.040734 |
| GO:0071559 | response to transforming growth factor beta | 0.15 | 0.027397 | 3 | 34 | 353 | 0.01558 | DPSC7.2 | 0.047505 |
| GO:0036473 | cell death in response to oxidative stress | 0.1 | 0.01531 | 2 | 19 | 108 | 0.035818 | DPSC7.2 | 0.082276 |
| GO:0070372 | regulation of ERK1 and ERK2 cascade | 0.1 | 0.017728 | 2 | 22 | 358 | 0.04701 | DPSC7.2 | 0.099361 |
| GO:0007059 | chromosome segregation | 0.344444 | 0.043411 | 31 | 56 | 488 | 9.92E-24 | DPSC7.7 | 3.91E-20 |
| GO:0098813 | nuclear chromosome segregation | 0.311111 | 0.034884 | 28 | 45 | 400 | 2.44E-23 | DPSC7.7 | 4.81E-20 |
| GO:0000819 | sister chromatid segregation | 0.288889 | 0.031008 | 26 | 40 | 281 | 2.31E-22 | DPSC7.7 | 2.29E-19 |
| GO:0140014 | mitotic nuclear division | 0.333333 | 0.043411 | 30 | 56 | 394 | 2.33E-22 | DPSC7.7 | 2.29E-19 |
| GO:0000070 | mitotic sister chromatid segregation | 0.277778 | 0.028682 | 25 | 37 | 228 | 3.94E-22 | DPSC7.7 | 3.11E-19 |
| GO:0007091 | metaphase/anaphase transition of mitotic cell cycle | 0.155556 | 0.011628 | 14 | 15 | 70 | 3.39E-16 | DPSC7.7 | 1.91E-13 |
| GO:0044784 | metaphase/anaphase transition of cell cycle | 0.155556 | 0.011628 | 14 | 15 | 73 | 3.39E-16 | DPSC7.7 | 1.91E-13 |
| GO:0051304 | chromosome separation | 0.166667 | 0.013953 | 15 | 18 | 116 | 9.81E-16 | DPSC7.7 | 4.84E-13 |
| GO:0002449 | lymphocyte mediated immunity | 0.044444 | 0.015504 | 4 | 20 | 449 | 0.045298 | DPSC7.7 | 0.09631 |
| GO:0002495 | antigen processing and presentation of peptide antigen via MHC class II | 0.044444 | 0.015504 | 4 | 20 | 114 | 0.045298 | DPSC7.7 | 0.09631 |

**Supplementary Table S13.** Table of gene ontology pathway associated with pathways enriched in DPSC7.5, DPSC7.8, DPSC7.10, and DPSC7.11, which were primarily composed of cells in the Ctrl sample, related to Supplementary Figure S7D-S7G.

| **Geneset_id** | **Description** | **fg_freq** | **bg_freq** | **n_fg** | **n_bg** | **n_set** | **p_val** | **cluster** | **p_adj** |
| --- | --- | --- | --- | --- | --- | --- | --- | --- | --- |
| GO:0007059 | chromosome segregation | 0.227848 | 0.033778 | 18 | 43 | 488 | 4.79E-12 | DPSC7.5 | 4.84E-10 |
| GO:0098813 | nuclear chromosome segregation | 0.177215 | 0.024352 | 14 | 31 | 400 | 4.53E-10 | DPSC7.5 | 3.72E-08 |
| GO:0042116 | macrophage activation | 0.037975 | 0.005499 | 3 | 7 | 124 | 0.00672 | DPSC7.5 | 0.026738 |
| GO:0051592 | response to calcium ion | 0.063291 | 0.017282 | 5 | 22 | 160 | 0.009257 | DPSC7.5 | 0.034399 |
| GO:2001020 | regulation of response to DNA damage stimulus | 0.063291 | 0.019639 | 5 | 25 | 273 | 0.016125 | DPSC7.5 | 0.047532 |
| GO:0043406 | positive regulation of MAP kinase activity | 0.063291 | 0.020424 | 5 | 26 | 339 | 0.019013 | DPSC7.5 | 0.054259 |
| GO:1900744 | regulation of p38MAPK cascade | 0.025316 | 0.003142 | 2 | 4 | 53 | 0.02103 | DPSC7.5 | 0.058327 |
| GO:0071772 | response to BMP | 0.037975 | 0.010212 | 3 | 13 | 268 | 0.041889 | DPSC7.5 | 0.090171 |
| GO:0009612 | response to mechanical stimulus | 0.050633 | 0.018068 | 4 | 23 | 226 | 0.04933 | DPSC7.5 | 0.101862 |
| GO:0043547 | positive regulation of GTPase activity | 0.050633 | 0.018068 | 4 | 23 | 448 | 0.04933 | DPSC7.5 | 0.101862 |
| GO:0048661 | positive regulation of smooth muscle cell proliferation | 0.09434 | 0.011146 | 5 | 14 | 114 | 0.000166 | DPSC7.8 | 0.002568 |
| GO:0006022 | aminoglycan metabolic process | 0.075472 | 0.007166 | 4 | 9 | 254 | 0.000305 | DPSC7.8 | 0.003538 |
| GO:0045765 | regulation of angiogenesis | 0.132075 | 0.028662 | 7 | 36 | 493 | 0.000516 | DPSC7.8 | 0.005486 |
| GO:0060840 | artery development | 0.056604 | 0.004777 | 3 | 6 | 107 | 0.001298 | DPSC7.8 | 0.009905 |
| GO:0097187 | dentinogenesis | 0.037736 | 0.001592 | 2 | 2 | 7 | 0.001748 | DPSC7.8 | 0.011722 |
| GO:0061448 | connective tissue development | 0.09434 | 0.018312 | 5 | 23 | 294 | 0.002085 | DPSC7.8 | 0.013467 |
| GO:0002053 | positive regulation of mesenchymal cell proliferation | 0.037736 | 0.002389 | 2 | 3 | 25 | 0.005103 | DPSC7.8 | 0.021665 |
| GO:0060337 | type I interferon signaling pathway | 0.056604 | 0.01035 | 3 | 13 | 115 | 0.015039 | DPSC7.8 | 0.046266 |
| GO:0051767 | nitric-oxide synthase biosynthetic process | 0.037736 | 0.003981 | 2 | 5 | 21 | 0.016104 | DPSC7.8 | 0.047505 |
| GO:0000302 | response to reactive oxygen species | 0.09434 | 0.031051 | 5 | 39 | 262 | 0.021322 | DPSC7.8 | 0.058603 |
| GO:0098813 | nuclear chromosome segregation | 0.229885 | 0.02795 | 20 | 36 | 400 | 1.35E-15 | DPSC7.10 | 5.49E-13 |
| GO:0000819 | sister chromatid segregation | 0.218391 | 0.024845 | 19 | 32 | 281 | 1.39E-15 | DPSC7.10 | 5.49E-13 |
| GO:0007059 | chromosome segregation | 0.252874 | 0.035714 | 22 | 46 | 488 | 2.73E-15 | DPSC7.10 | 8.28E-13 |
| GO:0000070 | mitotic sister chromatid segregation | 0.183908 | 0.020963 | 16 | 27 | 228 | 3.41E-13 | DPSC7.10 | 5.38E-11 |
| GO:0140014 | mitotic nuclear division | 0.229885 | 0.034938 | 20 | 45 | 394 | 3.66E-13 | DPSC7.10 | 5.56E-11 |
| GO:0006323 | DNA packaging | 0.137931 | 0.017857 | 12 | 23 | 382 | 3.14E-09 | DPSC7.10 | 2.21E-07 |
| GO:0051304 | chromosome separation | 0.091954 | 0.009317 | 8 | 12 | 116 | 1.26E-07 | DPSC7.10 | 7.08E-06 |
| GO:0034508 | centromere complex assembly | 0.08046 | 0.006988 | 7 | 9 | 75 | 1.64E-07 | DPSC7.10 | 8.68E-06 |
| GO:0006260 | DNA replication | 0.149425 | 0.028727 | 13 | 37 | 421 | 2.36E-07 | DPSC7.10 | 1.15E-05 |
| GO:0051383 | kinetochore organization | 0.068966 | 0.005435 | 6 | 7 | 28 | 5.33E-07 | DPSC7.10 | 2.31E-05 |
| GO:0140014 | mitotic nuclear division | 0.207547 | 0.03236 | 11 | 41 | 394 | 2.90E-07 | DPSC7.11 | 1.38E-05 |
| GO:0000819 | sister chromatid segregation | 0.169811 | 0.02131 | 9 | 27 | 281 | 5.17E-07 | DPSC7.11 | 2.27E-05 |
| GO:0030509 | BMP signaling pathway | 0.075472 | 0.011839 | 4 | 15 | 248 | 0.002647 | DPSC7.11 | 0.015291 |
| GO:0070373 | negative regulation of ERK1 and ERK2 cascade | 0.056604 | 0.006314 | 3 | 8 | 84 | 0.003338 | DPSC7.11 | 0.017913 |
| GO:0007223 | Wnt signaling pathway, calcium modulating pathway | 0.037736 | 0.003157 | 2 | 4 | 40 | 0.009763 | DPSC7.11 | 0.034739 |
| GO:0043507 | positive regulation of JUN kinase activity | 0.037736 | 0.003157 | 2 | 4 | 96 | 0.009763 | DPSC7.11 | 0.034739 |
| GO:0043405 | regulation of MAP kinase activity | 0.09434 | 0.026046 | 5 | 33 | 443 | 0.010282 | DPSC7.11 | 0.035383 |
| GO:0072089 | stem cell proliferation | 0.056604 | 0.009471 | 3 | 12 | 136 | 0.01164 | DPSC7.11 | 0.038481 |
| GO:1901185 | negative regulation of ERBB signaling pathway | 0.037736 | 0.003946 | 2 | 5 | 61 | 0.015837 | DPSC7.11 | 0.047505 |
| GO:0046330 | positive regulation of JNK cascade | 0.037736 | 0.005525 | 2 | 7 | 175 | 0.031512 | DPSC7.11 | 0.076141 |

**Supplementary Table S14.** Table of official accession number of the raw data in the National Microbiology Data Center (NMDC). The NMDC raw data database (https://nmdc.cn/resource/ genomics/sra) can be accessed for data query, and the URL in the following list can directly obtain the data information.

| **Sample description** | **Submission number** | **Accession number** | **Biological sample number** | **Raw data number** | **URL** |
| --- | --- | --- | --- | --- | --- |
| DPSCs infected by C*. albicans* 1 | SUB1718082904326 | NMDC10018945 | NMDC20165131 | NMDC40056821 | https://nmdc.cn/resource/genomics/sra/detail/NMDC40056821 |
| DPSCs infected by *C. albicans* 2 | SUB1718082904326 | NMDC10018945 | NMDC20165131 | NMDC40056822 | https://nmdc.cn/resource/genomics/sra/detail/NMDC40056822 |
| DPSCs infected by *S. mutans* 1 | SUB1718082904326 | NMDC10018945 | NMDC20165132 | NMDC40056823 | https://nmdc.cn/resource/genomics/sra/detail/NMDC40056823 |
| DPSCs infected by *S. mutans* 2 | SUB1718082904326 | NMDC10018945 | NMDC20165132 | NMDC40056824 | https://nmdc.cn/resource/genomics/sra/detail/NMDC40056824 |
| DPSCs infected by *S. mutans* 3 | SUB1718082904326 | NMDC10018945 | NMDC20165132 | NMDC40056825 | https://nmdc.cn/resource/genomics/sra/detail/NMDC40056825 |
| DPSCs without infection 1 | SUB1718082904326 | NMDC10018945 | NMDC20165133 | NMDC40056826 | https://nmdc.cn/resource/genomics/sra/detail/NMDC40056826 |
| DPSCs without infection 2 | SUB1718082904326 | NMDC10018945 | NMDC20165133 | NMDC40056827 | https://nmdc.cn/resource/genomics/sra/detail/NMDC40056827 |
